# Oxidative Precipitation Synthesis of Calcium-Doped Manganese Ferrite Nanoparticles for Magnetic Hyperthermia

**DOI:** 10.3390/ijms232214145

**Published:** 2022-11-16

**Authors:** Sérgio R. S. Veloso, Raquel G. D. Andrade, Valéria Gomes, Carlos O. Amorim, Vítor S. Amaral, Verónica Salgueiriño, Paulo J. G. Coutinho, Paula M. T. Ferreira, Miguel A. Correa-Duarte, Elisabete M. S. Castanheira

**Affiliations:** 1 Physics Centre of Minho and Porto Universities (CF-UM-UP) and LaPMET Associate Laboratory, University of Minho, Campus de Gualtar, 4710-057 Braga, Portugal; 2 Centre of Chemistry (CQUM), University of Minho, Campus de Gualtar, 4710-057 Braga, Portugal; 3 Physics Department and CICECO, University of Aveiro, Campus de Santiago, 3810-193 Aveiro, Portugal; 4 CINBIO, Universidad de Vigo, 36310 Vigo, Spain; 5 Departamento de Física Aplicada, Universidad de Vigo, 36310 Vigo, Spain

**Keywords:** magnetic nanoparticles, superparamagnetism, citrate-stabilization, calcium ferrites, manganese ferrites, magnetic hyperthermia

## Abstract

Superparamagnetic nanoparticles are of high interest for therapeutic applications. In this work, nanoparticles of calcium-doped manganese ferrites (Ca*_x_*Mn_1−*x*_Fe_2_O_4_) functionalized with citrate were synthesized through thermally assisted oxidative precipitation in aqueous media. The method provided well dispersed aqueous suspensions of nanoparticles through a one-pot synthesis, in which the temperature and Ca/Mn ratio were found to influence the particles microstructure and morphology. Consequently, changes were obtained in the optical and magnetic properties that were studied through UV-Vis absorption and SQUID, respectively. XRD and Raman spectroscopy studies were carried out to assess the microstructural changes associated with stoichiometry of the particles, and the stability in physiological pH was studied through DLS. The nanoparticles displayed high values of magnetization and heating efficiency for several alternating magnetic field conditions, compatible with biological applications. Hereby, the employed method provides a promising strategy for the development of particles with adequate properties for magnetic hyperthermia applications, such as drug delivery and cancer therapy.

## 1. Introduction

Magnetic nanoparticles have been of high interest for several biomedical applications, such as drug and gene delivery [1], magnetic resonance imaging [2], and magnetic hyperthermia [3]. The iron oxides are the most widely used type of magnetic nanoparticles, owing to the high saturation magnetization, chemical stability, low cytotoxicity, and rather easy and scalable synthesis [4]. Particularly interesting is the case of nanoparticles of spinel ferrites, with a unit cell consisting of a cubic close-packed arrangement with 32 oxygen atoms, and 8 tetrahedral (A) and 16 octahedral (B) occupancies for cation doping and distribution, as by the general formula (M^2+^)^A^[Fe_2_^3+^]^B^O_4_^2−^, with the consequent tuning of their characteristic properties (e.g., optical, magnetic and dielectric) [4,5,6]. Particularly, the manganese ferrites’ outstanding magnetic properties, high transition temperature, and chemical stability render these particles suitable for several applications, including hyperthermia [7] and MRI [8]. In addition, manganese can be consumed in the amount of 0.67–4.99 mg, with a mean value of 2.21 mg/day [9], and recently the Mn^2+^ released concentration from 3 to 18 nm nanoparticles was demonstrated to be in the range of safe doses [10]. Yet, as its cytotoxicity is still of concern [11], other chemical compositions, such as ferrites doped with calcium, have recently been proposed [12,13,14].

When it comes to the suitable applications of these nanoparticles, stabilization is a major factor to consider, so as to ensure the biocompatibility and prevent particle aggregation. Among the several functional groups that can be employed, the citrate has been commonly used for biomedical applications [15,16,17,18], including in commercial iron oxide nanoparticles such as the MRI contrast agent VSOP C184 [19]. Here, the citric acid coordinates the iron oxide surface, leaving at least one carboxylic acid group exposed, thus rendering the surface hydrophilic and avoiding particle aggregation. However, despite being commonly employed as a post-synthesis surface modification [20], if used during the synthetic process, the citric acid molecules can affect both the nucleation and growth, modifying the crystallite size and oxidation degree of the final nanoparticles [15,21,22,23]. Thus, there has been a growing search for one-pot strategies to obtained well-defined stabilized particles, either employing hydrothermal [18] or co-precipitation methods [15]. Recently, a thermally assisted oxidative precipitation was demonstrated to be a suitable and scalable method for the production of spherical and anisotropic magnetic particles [17,24,25]. In general, the synthesis reaction mechanism is described by an alkalization reaction of ferrous ion to ferrous hydroxide, and consequent dehydration reaction of iron hydroxide and ferric oxyhydroxide (resulting from Fe(OH)_2_ oxidation) to magnetite [26], as follows:

(1) $${\mathrm{Fe}}^{2+}+2{\mathrm{OH}}^{-}\overset{}{\to }\mathrm{Fe}{\left(\mathrm{OH}\right)}_{2}$$

(2) $$3\mathrm{Fe}{\left(\mathrm{OH}\right)}_{2}+\frac{1}{2}O_{2}\overset{}{\to }\mathrm{Fe}{\left(\mathrm{OH}\right)}_{2}+2\mathrm{FeOOH}\;+{\;H}_{2}O$$

(3) $$\mathrm{Fe}{\left(\mathrm{OH}\right)}_{2}+2\mathrm{FeOOH}\overset{}{\to }{\mathrm{Fe}}_{3}O_{4}+2H_{2}O$$

The final size of these superparamagnetic magnetite nanoparticles, stabilized with citrate, was reported to be affected by precursor concentration, ionic strength and reaction time [26]. For instance, the partial oxidation of the initially formed Fe(OH)_2_ into ferric oxyhydroxide is driven by the O_2_ dissolved in water, which, together with the lower ionic strength, leads to larger particles. However, a longer reaction time was reported to decrease the size of the particles. Hence, in this work, we describe the one-pot synthesis of ferrites doped with manganese and/or calcium stabilized with citrate through thermally assisted oxidative precipitation, requiring neither post-synthesis stabilization nor calcination treatment. The effect of the calcium/manganese cation ratio on the nanoparticle morphology and microstructure was studied, as well as the resulting magnetic properties. Further, the suitability of these magnetic nanoparticles for magnetic hyperthermia was assessed for several conditions adequate for biological applications.

## 2. Results and Discussion

### 2.1. Nanoparticles Morphology

**Figure 1:**
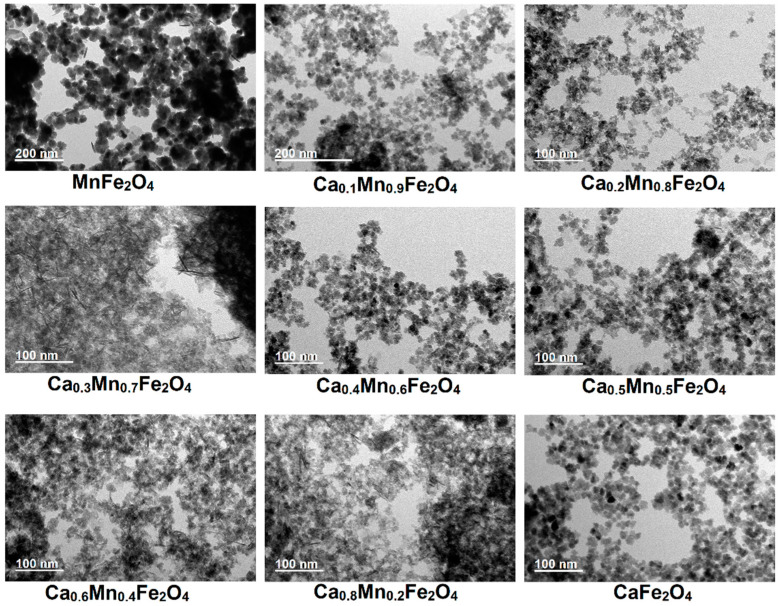
TEM images of the synthesized calcium and manganese-doped ferrites at 90 °C.

A transmission electron microscopy (TEM) analysis (Figure 1) demonstrated that increasing the calcium/manganese ratio affected the final morphology of the nanoparticles, narrowing the size distribution and decreasing the average size (from ~20 nm to ~5 nm). For instance, the presence of only manganese led to larger polydisperse particles, comprising single and multicore particles (more images of obtained structures are in Figure S1 in Supplementary Material). Doaga et al. [7] also reported for manganese doped ferrites that a higher content of manganese favoured the formation of larger particles obtained through co-precipitation method. The addition of calcium strongly favoured the formation of smaller and more monodisperse particles (Figure S2), but also displayed nanorod/spindle like structures. The formation of rods can be associated with the lepidocrocite or goethite as by-products of ferrous ion oxidation [27]. 

The multicore-like structures were mainly observed in the *x* = 0.4–0.6 calcium nominal fractions, and also in the *x* = 0.1 and *x* = 0.2 fractions, whereas a content of *x* = 0.3 and *x* = 0.8 presented a higher amount of small single core and rod-like structures. In the case of the samples synthesized with only calcium as dopant, (pseudo-)cubic and multicore-like particles were observed, and the sample was devoid of rod-like particles. The formation of multicore/flower-like particles was also reported by Gavilán et al. [28] from the oxidation of the Fe(OH)_2_ intermediate in water in the presence of dextran. 

The smaller size of the calcium (doped) ferrites can be associated with the prevention of crystal growth by Ca^2+^ as reported for other ferrites [29,30], in which the presence of citrate ions can provide additional electrostatic and steric repulsion, and thus hamper the fusing of the generated nuclei into larger particles. In addition, citrate is reported to not only prevent the oxidation of iron oxide to hematite [18], but also hamper the oxidation of Fe(II), as carboxylates are stronger complexing agents than the anions of simple ferrous salts [17], thus also delaying the nucleation. Nonetheless, the citrate role in ensuring a smaller size was clearly confirmed by the larger particle size obtained for manganese ferrites synthesized without citrate (Figure S3). In this sense, the presence of citrate is crucial to favor the repulsion between the initially formed nuclei, thus leading to the formation of smaller particles and also of multicore/flower-like particles, as the fusion between the formed cores is prevented.

**Figure 2:**
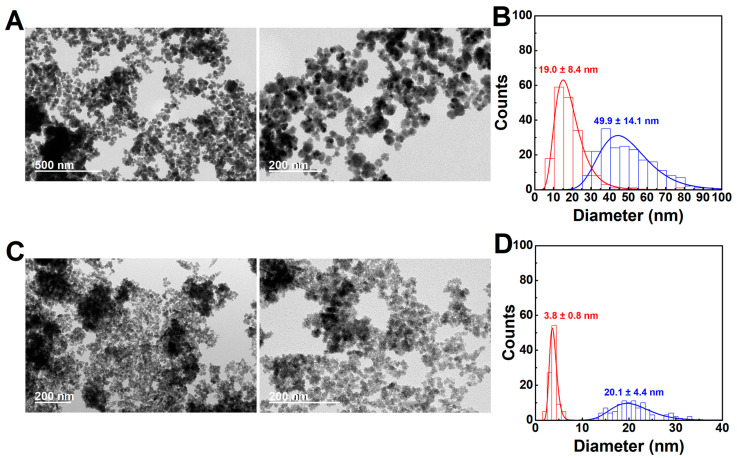
TEM images and histograms of the particles obtained from the synthesis of (**A**,**B**) manganese and (**C**,**D**) calcium ferrites at 100 °C, in the presence of citric acid. The red histogram corresponds to the smaller single cores forming part of the multicore-like particles (blue histogram).

Considering that the stabilization by citrate results from the chemical bonds between carboxyl groups of citrate and OH groups of the magnetic nanoparticles surface [15], a higher reaction temperature was tested to assess the impact of an increased chemical reaction rate. As displayed in Figure 2, a higher temperature (100 °C) led to samples devoid of rod-like structures and a predominance of the multicore particles, in both stoichiometric manganese and calcium ferrites. The formation of smaller single cores of the magnetic nanoparticles was noticed in the case of the calcium ferrite, which decreased from ~7 nm to ~4 nm, but not in the case of the manganese ferrite, which kept the same average size (~19 nm). Both samples displayed a large content of multicore particles with average sizes of ~50 nm and ~20 nm for the manganese and calcium ferrite, respectively. Summarizing this part, the thermally assisted oxidative precipitation in the presence of citrate and at 100 °C offers control over the production of multicore/flower-like nanoparticles, with the dopant ion playing a role in determining the final size of the small single cores. Nonetheless, the results are also promising for future research in the exploration of other reaction conditions, including the oxidant, as a means to assess other morphologies or tune the ones here described.

### 2.2. Microstructure Characterization

#### 2.2.1. X-ray Diffraction Analysis

**Figure 3:**
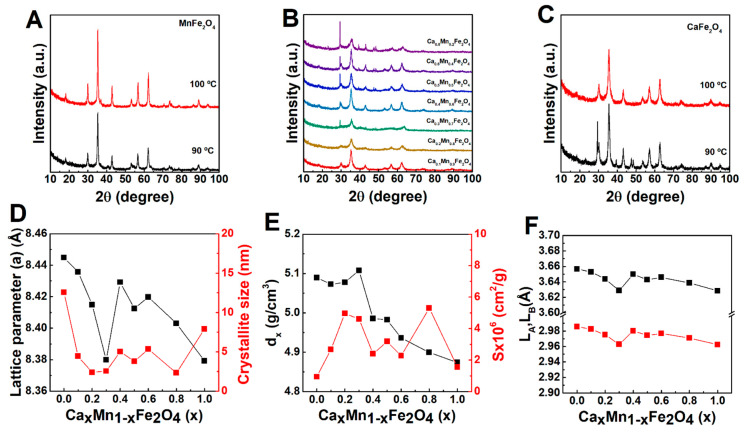
XRD patterns of (**A**) manganese ferrites, (**B**) calcium and manganese mixed ferrites, and (**C**) calcium ferrites. Dependence of (**D**) lattice parameter, crystallite size, (**E**) X-ray density (d_x_), specific surface area (S), and (**F**) hopping length in tetragonal (L_A_) and octahedral (L_B_) sites on the Ca^2+^ and Mn^2+^ content.

X-ray diffraction (XRD) studies were carried out to determine the phase composition and crystalline domain size of the nanoparticles, as well as to understand the changes observed in the particle size. The XRD patterns in Figure 3A–C revealed that all synthesized particles displayed crystalline nature, which endows the employed method suitable to achieve crystalline manganese and/or calcium ferrite nanoparticles, as other synthesis routes of calcium-doped ferrites require calcination after synthesis [14,29,30]. In all the cases, the patterns displayed Bragg’s reflections characteristic of the Fd3m space group, that can be indexed as (1 1 1), (2 2 0), (3 1 1), (4 2 0), (5 1 1), (4 4 0), and (6 2 0), reflecting the presence of manganese/calcium ferrites with a cubic structure [31,32,33]. Yet, another phase was formed in the samples with a larger calcium content (*x* > 0.2), with peaks indexed as (1 0 4), (1 1 0), (1 1 3), (2 0 2), (0 2 4), (0 1 8) and (1 1 6), associated with the presence of calcite (calcium carbonate) rhombohedral structure belonging to the R3c space group. Thus, the results also suggest that the obtained rods are a shape by-product of the reaction, and not lepidocrocite or goethite.

The synthesis of both stoichiometric manganese and calcium ferrites at 100 °C led to single-phase nanoparticles, suggesting that the calcium carbonate present in the mixed ferrites can be an intermediate of the reaction that is not completed at 90 °C. In this sense, the results suggest that Ca^2+^ might interfere with the nucleation and growth of the crystals, with a fraction of the iron ions remaining in an amorphous phase (mostly for *x* = 0.3 in a Ca*_x_*M_1−*x*_Fe_2_O_4_ ferrite). For instance, the formation of calcium carbonate has also been reported by Gomes et al. [34] in a sol-gel synthesis method, in which a single phase of CaFe_2_O_4_ could be achieved through calcination at 1000 °C.

The Rietveld refinement method was employed to assess the crystallographic properties of the several synthesised particles (see the refinement of each profile in Figure S4). As displayed in Figure 3D, the replacement of Mn^2+^ (0.80 Å) with Ca^2+^ (0.99 Å) led to a decrease of the average crystallite size, which is in agreement with TEM results. Hashhash et al. [35] suggested that a decrease of the particle size with Ca^2+^ content could be associated with the reaching of the ion’s solubility limit, which leads to its accumulation in the grain boundaries, thus suppressing the grain growth. In addition, the lattice parameter *a* also decreased, though an average increased value was obtained at *x* = 0.4. This behaviour can be associated with the occupation of the B-sites by Ca^2+^ ions, that have a larger ionic radius [35]. However, at *x* = 0.4, the migration of Ca^2+^ from B to A-sites might be favoured, promoting the migration of the smaller Mn^2+^ or Fe^3+^ ions to the B-sites. Chhaya et al. [36] reported that migration of Ca^2+^ to the A-sites replaces the smaller Fe^3+^ (0.64 Å), while a further increase might lead to a continuous reduction of Ca^2+^ ions in the A-sites and consequent reduction of lattice constant. Besides, Islam et al. [8] also reported that for a larger particle size the Mn^2+^ tended to occupy the A-sites. In this way, the hopping lengths in tetrahedral (L_A_) and octahedral (L_B_) sites displayed a decreasing trend with the Ca^2+^ content (except at *x* = 0.4), which was also obtained for the particles synthesized at 100 °C (see Table S1 in Supplementary Material). Further, for a low Ca^2+^ content (*x* < 0.4), the change in the molecular weight is not significant, so the decrease of the cell unit volume leads to an increase of the X-ray density and specific surface area, while both fell for larger Ca^2+^ content, except at *x* = 0.8, in which the concomitant reduction of crystallite size led to a larger specific surface area. Other parameters were calculated (Table S2), including the tetrahedral and octahedral radii, that further suggest the discussed transfer of Ca^2+^, Mn^2+^ and Fe^3+^ between A- and B-sites. The preferential occupation of the B-sites by Ca^2+^ and the formation of inverse spinels was also confirmed through estimation of the cation distribution using the Bertaut method (see discussion and Table S3 in Supplementary Material) [37].

#### 2.2.2. Raman Spectroscopy Characterization

**Figure 4:**
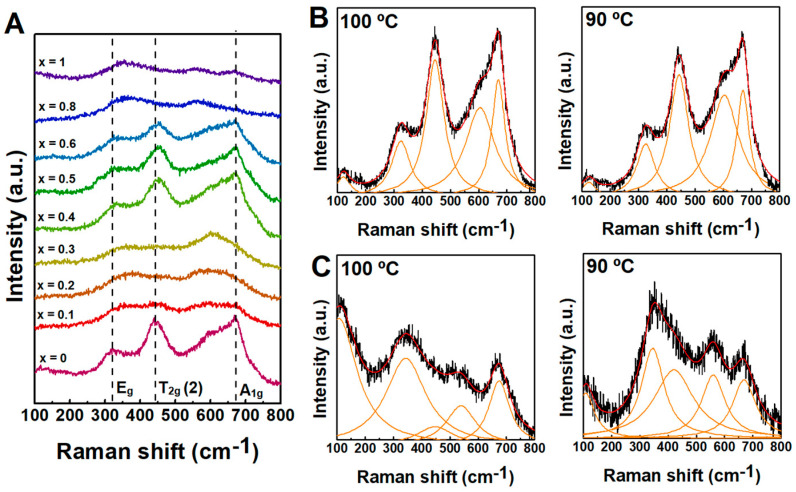
(**A**) A comparison of the Raman scattering spectra of the manganese- and/or calcium-doped ferrites obtained at 90 °C. The fitting of Lorentzian bands to Raman spectra of (**B**) manganese and (**C**) calcium ferrites synthesised at 90 °C and 100 °C.

Raman spectroscopy studies were carried out to get further insight into microstructural changes of the nanoparticles. A comparison of the several samples of nanoparticles obtained through synthesis at 90 °C is displayed in Figure 4, and the deconvolution with Lorentzian curves is included in Figure S5 (Supplementary Material). The samples did not display the calcite bands, which are commonly observed at ~150 cm^−1^, 280 cm^−1^ and 712 cm^−1^ [38], possibly for being below the threshold of the device sensitivity. The group theory predicts five Raman active modes for spinels with Fd3m space group (A_1g_ + E_g_ + 3T_2g_ bands), associated with the motion of O ions in the A and B-sites [39,40,41,42]. In general, when considering nanoparticles, the A_1g_, T_2g_(2), and E_g_ Raman modes are the most intense, located in this case around ~670 cm^−1^, ~450 cm^−1^, and ~320 cm^−1^, respectively (values reported in Table S4), which closely match other reported values for manganese-doped ferrites [43,44]. Besides, the spectra displayed some resemblance to profiles commonly observed in inverted spinels [45]. Particularly, the A_1g_ is associated with the symmetric stretching of O ions with respect to the metal ion in A-sites, which occurs together with the deformation of three metal-oxygen bonds at the octahedral sites (as the tetrahedral sites are not isolated) [40], thus being sensitive to the mass of the tetrahedral cation and size of the nanoparticles [46]. Hence, the splitting of the A_1g_ mode in two modes, as displayed in Figure 4B,C, can be associated to the fact of having two different cations in A and/or B sites. In addition to the metal-doping associated changes, the increase of Ca^2+^ was accompanied by a decrease of particle size, which is also suggested by an overall downshift, broadening of the Raman bands and intensity decrease resulting from the increase of micro-deformation and/or the dominance of phonon confinement [39,46]. Further, the incremental Ca^2+^ content induced changes also displayed some relation with the variation of lattice parameter (see further discussion in Supplementary Material) that further confirmed the ions distribution in both A and B-sites.

### 2.3. Optical Properties

The nanoparticles optical properties also displayed changes associated with the synthesis temperature and Ca/Mn ratio. Regarding the latter, the UV-vis absorbance was observed to achieve an enhanced absorbance in the near-infrared region at larger Mn^2+^ content, which has been commonly reported for manganese-doped ferrites [47].

The Tauc plot was further employed (see plots in Figure S6 in Supplementary Material) to estimate the optical direct band gap, as also described for other ferrites [48,49]. Here, the increased content of Mn^2+^ ions was accompanied by a red-shift of the optical band gap, which was found to decrease by synthesizing the particles at 100 °C. The red-shift of calcium ferrites band gap has also been reported for doping with Co^2+^ [49], which was associated with the increased particle size leading to a larger distance between atoms, and thus a reduction of the potential energy of materials’ electrons. In the work by Samira et al. [47], the substitution of Ni by Mn also produced a red-shift of the optical band gap, which was associated to Mn inducing inner bands that provided additional paths between the conduction and valence bands, producing a decrease of the band gap value.

In this sense, the results suggest that both the Ca/Mn ratio and synthesis temperature affect the optical band gap. Thus, both parameters can be employed to tune the specific capacitance that is commonly larger for smaller band gaps [47] and the energy harvesting ability of the nanoparticles, which can be of interest for photocatalysis applications in environmental cleaning.

### 2.4. Hydrodynamic Diameter and Zeta Potential

**Figure 5:**
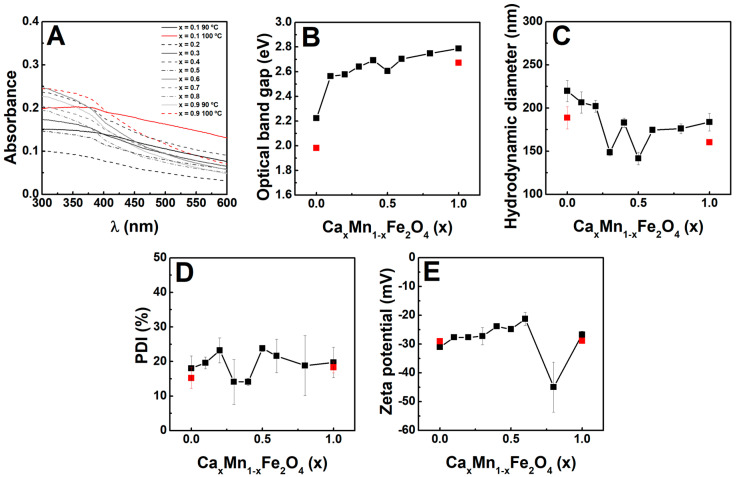
(**A**) UV-vis absorption spectra of the synthesized nanoparticles at 0.01 mg/mL and (**B**) dependence of the optical band gap on the Ca/Mn content for the particles synthesized at 90 °C (black squares) and 100 °C (red squares). The dependence of (**C**) hydrodynamic diameter, (**D**) polydispersity index (PDI), and (**E**) zeta potential on the particle type at 0.01 mg/mL is also included.

The behaviour of nanoparticles in the solution was studied at a concentration of 0.01 mg/mL. In agreement with the TEM and XRD results, the increasing content of Ca^2+^ ions led to a decrease of the hydrodynamic diameter (Figure 5C), which was also decreased by carrying out the synthesis at 100 °C. The obtained hydrodynamic diameter was obviously larger than the physical particle size, which can be associated with the high stability of the nanoparticles, as resulting from the electrostatic double layer stabilizing the nanoparticles in the solution or the formation of aggregates [50]. Indeed, this increased stability is also suggested by the rather low polydispersity index (in the ~0.1–0.2 range, Figure 5D), which might result from the particles dispersing into quasi-monodisperse structures, as also pointed out by the correlograms single decay in the majority of the samples (see Figure S7 in Supplementary Material). Further proof of the stability is confirmed by the highly negative zeta potential (Figure 5E) associated with the citrate functionalization at the nanoparticles surface, and correlated with the lack of noise (absence of sedimentation) in the correlograms.

In line with these results, the stabilization with citrate has been demonstrated to endow nanoparticles with good colloidal stability and circulation time, very appropriate for the potential bio-related applications [51,52]. Though a protein corona is commonly formed upon contact of the nanoparticles with the biologic fluids, which can influence the particle size and, consequently, the biodistribution in vivo and blood-clearance [53,54], the magnetic hyperthermia (vide infra) ability should not become substantially affected. Besides, the citrate negative charge provides a means for the adsorption and delivery of chemotherapeutic drugs through electrostatic interactions, such as doxorubicin [55], rendering the particles are a versatile platform for further developments in biomedical applications.

### 2.5. Magnetic Properties

**Figure 6:**
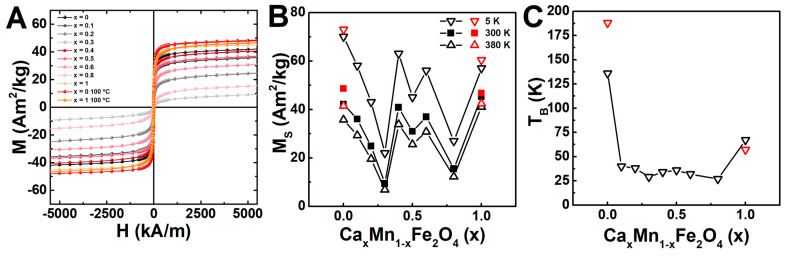
(**A**) Magnetization dependence on the applied magnetic field obtained for the manganese- and/or calcium-doped ferrites obtained at 90 °C (from *x* = 0 to *x* = 1) and 100 °C (*x* = 0 and *x* = 1) measured at 300 K. Dependence of the (**B**) saturation magnetization and (**C**) blocking temperature on the Ca/Mn ratio obtained from ZFC and FC curves under a field of 100 Oe.

Figure 6A includes the field-dependent magnetization of the different samples of Ca-doped manganese ferrite nanoparticles, offering relatively large values of saturation magnetization and negligible values of coercivity and remanence, at 300 K (see also Figure S8 and Table S5), reflecting superparamagnetic behaviour in all cases [56]. The largest values of saturation magnetization were registered for the stoichiometric manganese or calcium ferrite synthesised at 100 °C, stemming from the distribution of the magnetic cations (Mn^2+^ and Fe^3+^) in the crystalline structure (Figure 6B). The reproducibility of the method was also evidenced by the similarity of the stoichiometric manganese ferrite magnetic properties to other reported particles for the same synthesis method [57]. 

Regarding the particles with intermediary composition, the saturation magnetization displayed a Ca/Mn ratio dependence profile similar to the obtained in the crystallite and particle size, in which the obtained magnetization decreases for smaller sizes. This effect for individual particles is usually ascribed to the formation of a dead layer inversely proportional to the particle size, in which the spin canting and surface disorder (in-homogeneities, oxidation, and truncation of the crystalline lattice) are detrimental for the saturation magnetization [18,58,59]. However, this effect is very small and alone is not enough to explain the results, as the saturation magnetization remained reasonably high in some mixed ferrites, such as in the range *x* = 0.4 to 0.6, despite displaying smaller particle size than the CaFe_2_O_4_ and MnFe_2_O_4_ particles. Instead, as these samples were observed to display a cluster like morphology, in which close contacts are formed between the nanoparticles in each cluster, the minimization of the magnetic dead layer effect can be associated with the promotion of direct exchange and dipolar interactions that are described to minimize the decrease of the saturation magnetization [59]. Nonetheless, the inversion degree and inter-sublattice A-B super-exchange interaction were found to well describe the saturation magnetization dependence on the Ca/Mn ratio (see discussion and Figure S9A,B in Supplementary Material) [4,32,33,60,61,62,63,64,65].

In addition to the size and dipolar interactions between the clusters’ nanoparticles, the ferrites magnetocrystalline anisotropy plays a huge role in the final magnetic behavior, as evidenced in the measurements at 5 K, in which the coercivity displayed a Ca^2+^ content dependence profile (Figure S9C), in line with the pointed-out distribution of magnetic cations.

The magnetic properties were further characterized through zero field cooled (ZFC) and field cooled (FC) measurements under an applied field of 100 Oe. The ZFC-FC magnetization curves are displayed in Figure S10 in Supplementary Materials. The broader ZFC curves suggested larger particle size dispersity [4], which is mainly noticeable in the samples with *x* = 0 and in agreement with the TEM results. Nevertheless, the dipolar interactions between nanoparticles in the final flower-like structures should be taken into account as well [59], since this is suggested by the FC magnetization curve flatness observed in the low temperature range (mainly in the samples range from *x* = 0.4 to *x* = 1) [66,67]. Figure 6 includes the blocking temperature dependence on Ca/Mn ratio, obtained from the ZFC derivative (*dM/dT*, shown in Figure S10) [68], reflecting the highest values for the manganese ferrites. Furthermore, the obtained blocking temperatures for CaFe_2_O_4_ and MnFe_2_O_4_ particles were similar to other reported particles [69,70], proving therefore the reproducibility of the method. Overall, the magnetic characterization proves that the nanoparticles of CaFe_2_O_4_, MnFe_2_O_4_ and of intermediary formulations (*x* = 0.4 to 0.6) are superparamagnetic at room temperature and therefore very promising for magnetic targeting applications, given the large values of saturation magnetization.

### 2.6. Magnetic Hyperthermia

**Figure 7:**
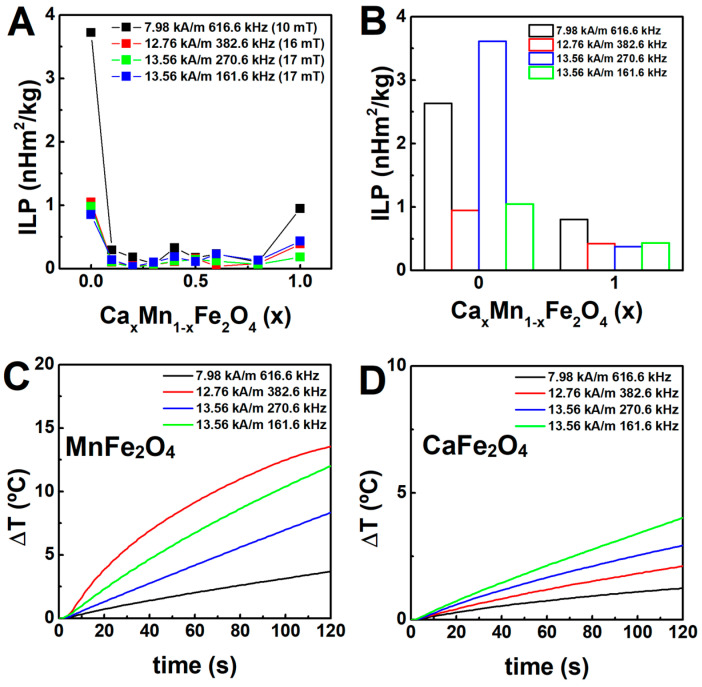
(**A**) Dependence of the intrinsic loss power (ILP) on the composition of the nanoparticles at 5 mg/mL. (**B**) ILP and (**C**,**D**) temperature variation over time of the manganese and calcium-doped ferrites obtained through synthesis at 100 °C. The equivalent magnetic flux density is also indicated in brackets.

The heating efficiency of the nanoparticles was evaluated considering alternating magnetic fields (AMF) of different amplitude and frequency, but fulfilling the medical threshold limit of $H_{0}f\;$≤ 5 × 10^9^ A m^−1^ s^−1^ [71]. The heating profiles are displayed in Figure 7.

In general, the incremental content of Ca^2+^ ions (in substitution of Mn^2+^ ions) led to a decrease of the heating efficiency (Figure 7A) that was quantitatively evaluated through the intrinsic loss power (ILP). However, a slight increase was obtained in the range *x* = 0.4 to *x* = 0.6, given the particular distribution of magnetic cations in the spinel ferrite crystalline structure for these stoichiometries. Furthermore, the samples synthesized at 100 °C displayed similar or slightly improved heating efficiency (Figure 7B) than the batch obtained at 90 °C, owing to the absence of phase impurities. Both samples of nanoparticles considered in this analysis induced a rather large temperature variation in 2 min (~15 °C when applying a field of ~13 kA/m (17 mT) and 382.6 kHz, in the case of the manganese ferrite, and ~4 °C with a field of ~13 kA/m (17 mT) and 161.6 kHz, for the calcium ferrite, of which mild hyperthermia treatments can take advantage of, in a short period of time.

Compared to other reported magnetic nanoparticles, the developed CaFe_2_O_4_ and MnFe_2_O_4_ particles displayed similar or improved heating efficiency than other transition metal-doped [72], cobalt-doped [73,74], calcium-doped [75], manganese-doped [8], and multicore particles [76].

Hence, the high heating efficiency of the manganese- and calcium-doped ferrites, together with the superparamagnetic behaviour, endow the developed particles suitable for magnetic hyperthermia therapy. Nonetheless, the exploration of several synthesis parameters, including reaction time, salts/reagents/ligand concentration, and reducing agents, are envisioned as future works to fine-tune the shape and heating performance of the developed particles.

## 3. Materials and Methods

### 3.1. Synthesis of Magnetic Nanoparticles

Citrate-stabilized iron oxide nanoparticles doped with calcium and/or manganese were synthesized through an oxidative precipitation method adapted from [26]. In general, trisodium citrate dehydrate (1 mmol) and NaOH (4 mmol) were added to 19 mL of ultrapure water at 90 °C or 100 °C. A 1 mL aqueous solution of FeSO_4_.7H_2_O (1.33 mmol) and the doping metal salt (MnSO_4_.H_2_O, Ca(CH_3_CO_2_)_2_) (0.66 mmol) was added, drop by drop, into the mixture under vigorous agitation and air open. After 2 h, the solution was cooled down to room temperature, washed through magnetic decantation with water/ethanol 1:1, and dried at 80 °C.

### 3.2. General Spectroscopic Methods

Absorption spectra were recorded in a Shimadzu UV-3600 Plus UV-Vis-NIR spectrophotometer (Shimadzu Corporation, Kyoto, Japan). A conventional PAN’alytical X’Pert PRO diffractometer (Malvern Panalytical Ltd., Malvern, UK) was used for X-ray diffraction (XRD) analyses, operating with Cu K_α_ radiation, in a Bragg-Brentano configuration, at the Electron Microscopy Unit, University of Trás-os-Montes and Alto Douro (UTAD), Vila Real, Portugal. Raman spectroscopy measurements were performed at room temperature with a Renishaw inVia Reflex Raman confocal microscope system (Wotton-under-Edge, Stroud, UK), equipped with a high-resolution grating of 1200 grooves mm^−1^. The excitation line, 785 nm, of a NIR diode laser was focused onto the sample by a ×20 objective with a numerical aperture (NA) value of 0.40 in a backscattering geometry. The spectra were acquired with a measured power of about 650 µW on the sample, with a spectral acquisition time of 120 s over one accumulation and the range 100–1000 cm^−1^. The average hydrodynamic diameter and zeta potential of the nanoparticles (n = 3 independent runs) were measured in phosphate buffer pH 7.4 at 0.01 mM in a Dynamic Light Scattering (DLS) equipment Litesizer^TM^ 500 from Anton Paar (Anton Paar GmbH, Graz, Austria), using a semiconductor laser diode of 40 mW and λ = 658 nm, backscatter angle (175°), and a controlled temperature of 25 °C.

### 3.3. Transmission Electron Microscopy (TEM)

TEM images of nanoparticles were recorded using a high contrast JEOL JEM-1010, operating at 100 kV (CACTI, Vigo, Spain). A small portion of the sample was placed onto a TEM 400 mesh copper grid with Formvar/Carbon (ref. S162-4 from Agar Scientific), held by tweezers and the excess solution was cleaned. The processing of STEM images was performed using ImageJ software (National Institutes of Health, NIH, Bethesda, MD, USA), which consisted in enhancing local contrast and adjusting brightness followed by manual selection of fibres.

### 3.4. Magnetic Properties

Magnetic measurements were performed in an MPMS3 SQUID magnetometer (Quantum Design Inc., San Diego, CA, USA). The field-dependent magnetization (hysteresis cycles) of the samples were measured in the large field range (up to H = 5570.42 kA/m or B = 7 T) for each sample. In all the cases at 5 K, 300 K, and 380 K, given the room temperature applications they are designed for, a specific magnetic field correction for the trapped flux in the superconducting coil was conducted, achieving an accuracy of residual less than 0.16 kA/m [77].

### 3.5. Hyperthermia Measurements

With the aim of evaluating the heating performance, magneto-caloric measurements were carried out using a hyperthermia system magneTherm (nanoTherics, Warrington, UK), working at f ≈ (162, 271, 383, 617) kHz and at the magnetic field H = (13.56, 12.76, 7.98) kA/m. For all experiments, the initial temperature was stabilized before starting the measurement. Next, the AC magnetic field was applied for 10 min, and the temperature variation was recorded using a thermocouple.

## 4. Conclusions

In this work, citrate-stabilized multicore nanoparticles of Ca-doped manganese ferrite (Ca*_x_*Mn_1−*x*_Fe_2_O_4_) were synthesized through a thermally assisted oxidative precipitation in aqueous media. While the incremental content of calcium was found to decrease the average nanoparticle size, decreasing the synthesis temperature from 100 to 90 °C was accompanied by the formation of an impurity phase of calcite.

Regarding the resulting particle shape, the synthesis resulted in the formation of single, multicore and rod-like particles, but the synthesis carried out at 100 °C allowed to preferentially obtain the multicore nanoparticles. Furthermore, the variation in the Mn/Ca ratio led to changes in the microstructure, mainly in the lattice parameter and cation distribution, and consequently in the final magnetic properties.

The particles displayed great colloidal stability, with a low podydispersity, lack of sedimentation and highly negative zeta potential.

The obtained calcium-doped manganese ferrite nanoparticles displayed relatively large values of saturation magnetization and heating efficiency, higher than other reported superparamagnetic nanoparticles, which, together with the superparamagnetic behaviour, render them suitable for therapeutic applications, such as drug delivery and cancer therapy.

## Data Availability

Not applicable.

## Supplementary Materials

The following supporting information can be downloaded at: https://www.mdpi.com/article/10.3390/ijms232214145/s1.

## References

[1] Mitchell M.J., Billingsley M.M., Haley R.M., Wechsler M.E., Peppas N.A., Langer R. (2021). Engineering Precision Nanoparticles for Drug Delivery. Nat. Rev. Drug Discov..

[2] Caspani S., Magalhães R., Araújo J.P., Sousa C.T. (2020). Magnetic Nanomaterials as Contrast Agents for MRI. Materials.

[3] Liu X., Zhang Y., Wang Y., Zhu W., Li G., Ma X., Zhang Y., Chen S., Tiwari S., Shi K. (2020). Comprehensive Understanding of Magnetic Hyperthermia for Improving Antitumor Therapeutic Efficacy. Theranostics.

[4] Kurian J., Lahiri B.B., Mathew M.J., Philip J. (2021). High Magnetic Fluid Hyperthermia Efficiency in Copper Ferrite Nanoparticles Prepared by Solvothermal and Hydrothermal Methods. J. Magn. Magn. Mater..

[5] Gahrouei Z.E., Labbaf S., Kermanpur A. (2020). Cobalt Doped Magnetite Nanoparticles: Synthesis, Characterization, Optimization and Suitability Evaluations for Magnetic Hyperthermia Applications. Phys. E Low-Dimens. Syst. Nanostruct..

[6] Chand P., Vaish S., Kumar P. (2017). Structural, Optical and Dielectric Properties of Transition Metal (MFe_2_O_4_; M = Co, Ni and Zn) Nanoferrites. Phys. B Condens. Matter.

[7] Doaga A., Cojocariu A.M., Amin W., Heib F., Bender P., Hempelmann R., Caltun O.F. (2013). Synthesis and Characterizations of Manganese Ferrites for Hyperthermia Applications. Mater. Chem. Phys..

[8] Islam K., Haque M., Kumar A., Hoq A., Hyder F., Hoque S.M. (2020). Manganese Ferrite Nanoparticles (MnFe_2_O_4_): Size Dependence for Hyperthermia and Negative/Positive Contrast Enhancement in MRI. Nanomaterials.

[9] Tripathi R.M., Mahapatra S., Raghunath R., Kumar A.V., Sadasivan S. (2002). Daily Intake of Manganese by Adult Population of Mumbai, India. Sci. Total Environ..

[10] Ma H., Guo L., Zhang H., Wang Y., Miao Y., Liu X., Peng M., Deng X., Peng Y., Fan H. (2022). The Metal Ion Release of Manganese Ferrite Nanoparticles: Kinetics, Effects on Magnetic Resonance Relaxivities, and Toxicity. ACS Appl. Bio Mater..

[11] Zhang L., Xiao S., Kang X., Sun T., Zhou C., Xu Z., Du M., Zhang Y., Wang G., Liu Y. (2021). Metabolic Conversion and Removal of Manganese Ferrite Nanoparticles in Raw264.7 Cells and Induced Alteration of Metal Transporter Gene Expression. Int. J. Nanomed..

[12] Khanna L., Verma N.K. (2014). Biocompatibility and Superparamagnetism in Novel Silica/CaFe_2_O_4_ Nanocomposite. Mater. Lett..

[13] Noor A., Akhtar M.N., Khan S.N., Nazir M.S., Yousaf M. (2020). Synthesis, Morphological and Electromagnetic Evaluations of Ca Doped Mn Spinel Nanoferrites for GHz Regime Applications. Ceram. Int..

[14] Khanna L., Verma N.K. (2013). Size-Dependent Magnetic Properties of Calcium Ferrite Nanoparticles. J. Magn. Magn. Mater..

[15] Li L., Mak K.Y., Leung C.W., Chan K.Y., Chan W.K., Zhong W., Pong P.W.T. (2013). Effect of Synthesis Conditions on the Properties of Citric-Acid Coated Iron Oxide Nanoparticles. Microelectron. Eng..

[16] Poller W.C., Löwa N., Schleicher M., Münster-Wandowski A., Taupitz M., Stangl V., Ludwig A., Wiekhorst F. (2020). Initial Interaction of Citrate-Coated Iron Oxide Nanoparticles with the Glycocalyx of THP-1 Monocytes Assessed by Real-Time Magnetic Particle Spectroscopy and Electron Microscopy. Sci. Rep..

[17] Granath T., Mandel K., Löbmann P. (2021). Overcoming the Inhibition Effects of Citrate: Precipitation of Ferromagnetic Magnetite Nanoparticles with Tunable Morphology, Magnetic Properties, and Surface Charge via Ferrous Citrate Oxidation. Part. Part. Syst. Charact..

[18] Maurizi L., Bouyer F., Paris J., Demoisson F., Saviot L., Millot N. (2011). One Step Continuous Hydrothermal Synthesis of Very Fine Stabilized Superparamagnetic Nanoparticles of Magnetite. Chem. Commun..

[19] Boyer C., Whittaker M.R., Bulmus V., Liu J., Davis T.P. (2010). The Design and Utility of Polymer-Stabilized Iron-Oxide Nanoparticles for Nanomedicine Applications. NPG Asia Mater..

[20] Jawad A., Al-Abodi E.E. (2019). Investigating (Fe_3_O_4_) Magnetic Nanoparticles Impregnated onto Tri-Sodium Citrate to Remove, of Methylene Blue Dye from Aqueous Solutions. AIP Conf. Proc..

[21] Ur Rahman Z., Dong Y.L., Ren C., Zhang Z.Y., Chen X. (2012). Protein Adsorption on Citrate Modified Magnetic Nanoparticles. J. Nanosci. Nanotechnol..

[22] Saraswathy A., Nazeer S.S., Jeevan M., Nimi N., Arumugam S., Harikrishnan V.S., Varma P.R.H., Jayasree R.S. (2014). Citrate Coated Iron Oxide Nanoparticles with Enhanced Relaxivity for in Vivo Magnetic Resonance Imaging of Liver Fibrosis. Colloids Surf. B Biointerfaces.

[23] Cheraghipour E., Javadpour S., Mehdizadeh A.R. (2012). Citrate Capped Superparamagnetic Iron Oxide Nanoparticles Used for Hyperthermia Therapy. J. Biomed. Sci. Eng..

[24] Asimakidou T., Makridis A., Veintemillas-Verdaguer S., Morales M.P., Kellartzis I., Mitrakas M., Vourlias G., Angelakeris M., Simeonidis K. (2020). Continuous Production of Magnetic Iron Oxide Nanocrystals by Oxidative Precipitation. Chem. Eng. J..

[25] Granath T., Löbmann P., Mandel K. (2021). Oxidative Precipitation as a Versatile Method to Obtain Ferromagnetic Fe_3_O_4_ Nano- and Mesocrystals Adjustable in Morphology and Magnetic Properties. Part. Part. Syst. Charact..

[26] Hui C., Shen C., Yang T., Bao L., Tian J., Ding H., Li C., Gao H.-J. (2008). Large-Scale Fe 3 O 4 Nanoparticles Soluble in Water Synthesized by a Facile Method. J. Phys. Chem. C.

[27] Sleiman N., Deluchat V., Wazne M., Courtin A., Saad Z., Kazpard V., Baudu M. (2016). Role of Iron Oxidation Byproducts in the Removal of Phosphate from Aqueous Solution. RSC Adv..

[28] Gavilán H., Kowalski A., Heinke D., Sugunan A., Sommertune J., Varón M., Bogart L.K., Posth O., Zeng L., González-Alonso D. (2017). Colloidal Flower-Shaped Iron Oxide Nanoparticles: Synthesis Strategies and Coatings. Part. Part. Syst. Charact..

[29] Hirazawa H., Kusamoto S., Aono H., Naohara T., Mori K., Hattori Y., Maehara T., Watanabe Y. (2008). Preparation of Fine Mg1-XCaXFe2O4 Powder Using Reverse Coprecipitation Method for Thermal Coagulation Therapy in an Ac Magnetic Field. J. Alloys Compd..

[30] Pereira D.S.M., Cardoso B.D., Rodrigues A.R.O., Amorim C.O., Amaral V.S.A., Almeida B.G., Queiroz M.-J.R.P., Martinho O., Baltazar F., Calhelha R.C. (2019). Magnetoliposomes Containing Calcium Ferrite Nanoparticles for Applications in Breast Cancer Therapy. Pharmaceutics.

[31] Satalkar M., Kane S.N. (2016). On the Study of Structural Properties and Cation Distribution of Zn_0.75-x_Ni_x_Mg_0.15_Cu_0.1_Fe_2_O_4_ Nano Ferrite: Effect of Ni Addition. J. Phys. Conf. Ser..

[32] Nikam D.S., Jadhav S.V., Khot V.M., Bohara R.A., Hong C.K., Mali S.S., Pawar S.H. (2015). Cation Distribution, Structural, Morphological and Magnetic Properties of Co_1−x_Zn_x_Fe_2_O_4_ (x = 0–1) Nanoparticles. RSC Adv..

[33] Bamzai K.K., Kour G., Kaur B., Kulkarni S.D. (2014). Preparation, and Structural and Magnetic Properties of Ca Substituted Magnesium Ferrite with Composition MgCa_x_Fe_2−x_O_4_ (x = 0.00, 0.01, 0.03, 0.05, 0.07). J. Mater..

[34] Gomes H.C., Teixeira S.S., Graça M.P.F. (2022). Synthesis of Calcium Ferrite for Energy Storage Applications. J. Alloys Compd..

[35] Hashhash A., Kaiser M. (2021). Synthesis and Characterization of Calcium-Substituted Mg-Co-Cr Ferrite Nanoparticles with a Crystallite Size Less Than 10 Nm. J. Supercond. Nov. Magn..

[36] Chhaya S.D., Pandya M.P., Chhantbar M.C., Modi K.B., Baldha G.J., Joshi H.H. (2004). Study of Substitution Limit, Structural, Bulk Magnetic and Electrical Properties of Ca^2+^ Substituted Magnesium Ferrite. J. Alloys Compd..

[37] Weil L., Bertaut F., Bochirol L. (1950). Propriétés Magnétiques et Structure de La Phase Quadratique Du Ferrite de Cuivre. J. Phys. Radium.

[38] Donnelly F.C., Purcell-Milton F., Framont V., Cleary O., Dunne P.W., Gun’ko Y.K. (2017). Synthesis of CaCO_3_ Nano- and Micro-Particles by Dry Ice Carbonation. Chem. Commun..

[39] Massoudi J., Smari M., Nouri K., Dhahri E., Khirouni K., Bertaina S., Bessais L., Hlil E.K. (2020). Magnetic and Spectroscopic Properties of Ni-Zn-Al Ferrite Spinel: From the Nanoscale to Microscale. RSC Adv..

[40] Testa-Anta M., Ramos-Docampo M.A., Comesaña-Hermo M., Rivas-Murias B., Salgueiriño V. (2019). Raman Spectroscopy to Unravel the Magnetic Properties of Iron Oxide Nanocrystals for Bio-Related Applications. Nanoscale Adv..

[41] Wang Z., Schiferl D., Zhao Y., O’Neill H.S.C. (2003). High Pressure Raman Spectroscopy of Spinel-Type Ferrite ZnFe_2_O_4_. J. Phys. Chem. Solids.

[42] Freire R.M., Ribeiro T.S., Vasconcelos I.F., Denardin J.C., Barros E.B., Mele G., Carbone L., Mazzetto S.E., Fechine P.B.A. (2013). MZnFe_2_O_4_ (M = Ni, Mn) Cubic Superparamagnetic Nanoparticles Obtained by Hydrothermal Synthesis. J. Nanoparticle Res..

[43] Wang W., Ding Z., Zhao X., Wu S., Li F., Yue M., Liu J.P. (2015). Microstructure and Magnetic Properties of MFe_2_O_4_ (M = Co, Ni, and Mn) Ferrite Nanocrystals Prepared Using Colloid Mill and Hydrothermal Method. J. Appl. Phys..

[44] Nekvapil F., Bunge A., Radu T., Cinta Pinzaru S., Turcu R. (2020). Raman Spectra Tell Us so Much More: Raman Features and Saturation Magnetization for Efficient Analysis of Manganese Zinc Ferrite Nanoparticles. J. Raman Spectrosc..

[45] Babu K.V., Kumar G.V.S., Satyanarayana G., Sailaja B., Lakshmi C.C.S. (2018). Microstructural and Magnetic Properties of Ni_1−x_Cu_x_Fe_2_O_4_ (x = 0.05, 0.1 and 0.15) Nano-Crystalline Ferrites. J. Sci. Adv. Mater. Devices.

[46] Galinetto P., Albini B., Bini M., Mozzati M.C., Nascimento G. (2018). Raman Spectroscopy in Zinc Ferrites Nanoparticles. Raman Spectroscopy.

[47] Sharifi S., Yazdani A., Rahimi K. (2020). Incremental Substitution of Ni with Mn in NiFe_2_O_4_ to Largely Enhance Its Supercapacitance Properties. Sci. Rep..

[48] Baig M.M., Zulfiqar S., Yousuf M.A., Touqeer M., Ullah S., Agboola P.O., Warsi M.F., Shakir I. (2020). Structural and Photocatalytic Properties of New Rare Earth La^3+^ Substituted MnFe_2_O_4_ Ferrite Nanoparticles. Ceram. Int..

[49] Hashemi A., Naseri M., Ghiyasvand S., Naderi E., Vafai S. (2022). Evaluation of Physical Properties, Cytotoxicity, and Antibacterial Activities of Calcium–Cadmium Ferrite Nanoparticles. Appl. Phys. A Mater. Sci. Process..

[50] Jitkang L., Pin Y.S., Xin C.H., Chun L.S. (2013). Characterization of Magnetic Nanoparticle by Dynamic Light Scattering. Nanoscale Res. Lett..

[51] Nowak-Jary J., Machnicka B. (2022). Pharmacokinetics of Magnetic Iron Oxide Nanoparticles for Medical Applications. J. Nanobiotechnology.

[52] Iacovita C., Fizeșan I., Pop A., Scorus L., Dudric R., Stiufiuc G., Vedeanu N., Tetean R., Loghin F., Stiufiuc R. (2020). In Vitro Intracellular Hyperthermia of Iron Oxide Magnetic Nanoparticles, Synthesized at High Temperature by a Polyol Process. Pharmaceutics.

[53] Jedlovszky-Hajdú A., Bombelli F.B., Monopoli M.P., Tombácz E., Dawson K.A. (2012). Surface Coatings Shape the Protein Corona of SPIONs with Relevance to Their Application in Vivo. Langmuir.

[54] Yallapu M.M., Chauhan N., Othman S.F., Khalilzad-Sharghi V., Ebeling M.C., Khan S., Jaggi M., Chauhan S.C. (2015). Implications of Protein Corona on Physico-Chemical and Biological Properties of Magnetic Nanoparticles. Biomaterials.

[55] Nawara K., Romiszewski J., Kijewska K., Szczytko J., Twardowski A., Mazur M., Krysinski P. (2012). Adsorption of Doxorubicin onto Citrate-Stabilized Magnetic Nanoparticles. J. Phys. Chem. C.

[56] Caruntu D., Caruntu G., O’Connor C.J. (2007). Magnetic Properties of Variable-Sized Fe_3_O_4_ Nanoparticles Synthesized from Non-Aqueous Homogeneous Solutions of Polyols. J. Phys. D Appl. Phys..

[57] Veloso S.R.S., Silva J.F.G., Hilliou L., Moura C., Coutinho P.J.G., Martins J.A., Testa-Anta M., Salgueiriño V., Correa-Duarte M.A., Ferreira P.M.T. (2021). Impact of Citrate and Lipid-Functionalized Magnetic Nanoparticles in Dehydropeptide Supramolecular Magnetogels: Properties, Design and Drug Release. Nanomaterials.

[58] Karimi Z., Karimi L., Shokrollahi H. (2013). Nano-Magnetic Particles Used in Biomedicine: Core and Coating Materials. Mater. Sci. Eng. C.

[59] Otero-Lorenzo R., Fantechi E., Sangregorio C., Salgueiriño V. (2016). Solvothermally Driven Mn Doping and Clustering of Iron Oxide Nanoparticles for Heat Delivery Applications. Chem. A Eur. J..

[60] Ouyahia S., Rais A., Bozzo B., Taibi K., Addou A. (2020). Cations Distribution by Rietveld Refinement and Magnetic Properties of MgCr_x_Fe_2−x_O_4_ Spinel Ferrites. Appl. Phys. A Mater. Sci. Process..

[61] Sharma R., Thakur P., Kumar M., Barman P.B., Sharma P., Sharma V. (2017). Enhancement in A-B Super-Exchange Interaction with Mn Substitution in Mg-Zn Ferrites as a Heating Source in Hyperthermia Applications. Ceram. Int..

[62] Nitika, Rana A., Kumar V. (2021). Evaluation of Structural, Magnetic, Optical, Electrical, and Humidity Sensing Properties of Manganese-Substituted Zinc Ferrite Nanoparticles. Appl. Phys. A.

[63] Kumar P., Pathak S., Singh A., Jain K., Khanduri H., Wang L., Kim S.-K., Pant R.P. (2022). Observation of Intrinsic Fluorescence in Cobalt Ferrite Magnetic Nanoparticles by Mn^2+^ Substitution and Tuning the Spin Dynamics by Cation Distribution. J. Mater. Chem. C.

[64] Assar S.T., Abosheiasha H.F. (2015). Effect of Ca Substitution on Some Physical Properties of Nano-Structured and Bulk Ni-Ferrite Samples. J. Magn. Magn. Mater..

[65] Topkaya R., Baykal A., Demir A. (2013). Yafet–Kittel-Type Magnetic Order in Zn-Substituted Cobalt Ferrite Nanoparticles with Uniaxial Anisotropy. J. Nanopart. Res..

[66] Vargas J.M., Nunes W.C., Socolovsky L.M., Knobel M., Zanchet D. (2005). Effect of Dipolar Interaction Observed in Iron-Based Nanoparticles. Phys. Rev. B.

[67] Félix L.L., Rodriguez Martínez M.A., Pacheco Salazar D.G., Huamani Coaquira J.A. (2020). One-Step Synthesis of Polyethyleneimine-Coated Magnetite Nanoparticles and Their Structural, Magnetic and Power Absorption Study. RSC Adv..

[68] Bruvera I.J., Mendoza Zélis P., Pilar Calatayud M., Goya G.F., Sánchez F.H. (2015). Determination of the Blocking Temperature of Magnetic Nanoparticles: The Good, the Bad, and the Ugly. J. Appl. Phys..

[69] Yelenich O., Solopan S., Kolodiazhnyi T., Tykhonenko Y., Tovstolytkin A., Belous A. (2015). Magnetic Properties and AC Losses in AFe_2_O_4_ (A = Mn, Co, Ni, Zn) Nanoparticles Synthesized from Nonaqueous Solution. J. Chem..

[70] Khanna L., Verma N.K. (2013). PEG/CaFe_2_O_4_ Nanocomposite: Structural, Morphological, Magnetic and Thermal Analyses. Phys. B Condens. Matter.

[71] Mehdaoui B., Meffre A., Carrey J., Lachaize S., Lacroix L.-M., Gougeon M., Chaudret B., Respaud M. (2011). Optimal Size of Nanoparticles for Magnetic Hyperthermia: A Combined Theoretical and Experimental Study. Adv. Funct. Mater..

[72] Gupta R., Tomar R., Chakraverty S., Sharma D. (2021). Effect of Manganese Doping on the Hyperthermic Profile of Ferrite Nanoparticles Using Response Surface Methodology. RSC Adv..

[73] Aslibeiki B., Eskandarzadeh N., Jalili H., Ghotbi Varzaneh A., Kameli P., Orue I., Chernenko V., Hajalilou A., Ferreira L.P., Cruz M.M. (2022). Magnetic Hyperthermia Properties of CoFe_2_O_4_ Nanoparticles: Effect of Polymer Coating and Interparticle Interactions. Ceram. Int..

[74] Nasrin S., Chowdhury F.U.Z., Hoque S.M. (2019). Study of Hyperthermia Temperature of Manganese-Substituted Cobalt Nano Ferrites Prepared by Chemical Co-Precipitation Method for Biomedical Application. J. Magn. Magn. Mater..

[75] Kheradmand A., Vahidi O., Masoudpanah S.M. (2018). Magnetic, Hyperthermic and Structural Properties of Zn Substituted CaFe_2_O_4_ Powders. Appl. Phys. A Mater. Sci. Process..

[76] Lopes F.A.C., Fernandes A.V.F., Rodrigues J.M., Queiroz M.-J.R.P., Almeida B.G., Pires A., Pereira A.M., Araújo J.P., Castanheira E.M.S., Rodrigues A.R.O. (2022). Magnetoliposomes Containing Multicore Nanoparticles and a New Antitumor Thienopyridine Compound with Potential Application in Chemo/Thermotherapy. Biomedicines.

[77] Amorim C.O., Mohseni F., Dumas R.K., Amaral V.S., Amaral J.S. (2021). A Geometry-Independent Moment Correction Method for the MPMS3 SQUID-Based Magnetometer. Meas. Sci. Technol..

