# Supplementary material for: Oxidative Precipitation Synthesis of Calcium-Doped Manganese Ferrite Nanoparticles for Magnetic Hyperthermia
Source: Int J Mol Sci. 2022 Nov 16;23(22):14145. doi: 10.3390/ijms232214145 (PMC9695608; doi:10.3390/ijms232214145)
Supplement: Supplementary file 1 [file ijms-23-14145-s001.zip › ijms-2020205-supplementary.pdf]

Oxidative Precipitation Synthesis of Calcium-Doped Manganese Ferrite Nanoparticles for Magnetic Hyperthermia

Sérgio R. S. Veloso [1], Raquel G. D. Andrade [1], Valéria Gomes [1,2], Carlos O. Amorim [3], Vítor S. Amaral [3], Verónica Salgueiriño [4,5], Paulo J. G. Coutinho [1], Paula M. T. Ferreira [2], Miguel A. Correa-Duarte [4] and Elisabete M. S. Castanheira [1,*]

[1] Physics Centre of Minho and Porto Universities (CF-UM-UP) and LaPMET Associate Laboratory, University of Minho, Campus de Gualtar, 4710-057 Braga, Portugal
[2] Centre of Chemistry (CQUM), University of Minho, Campus de Gualtar, 4710-057 Braga, Portugal
[3] Physics Department and CICECO, University of Aveiro, Campus de Santiago, 3810-193 Aveiro, Portugal
[4] CINBIO, Universidad de Vigo, 36310 Vigo, Spain
[5] Departamento de Física Aplicada, Universidad de Vigo, 36310 Vigo, Spain
* Correspondence: ecoutinho@fisica.uminho.pt; Tel.: +351-253-604-321

Supplementary Material

Nanoparticles morphology

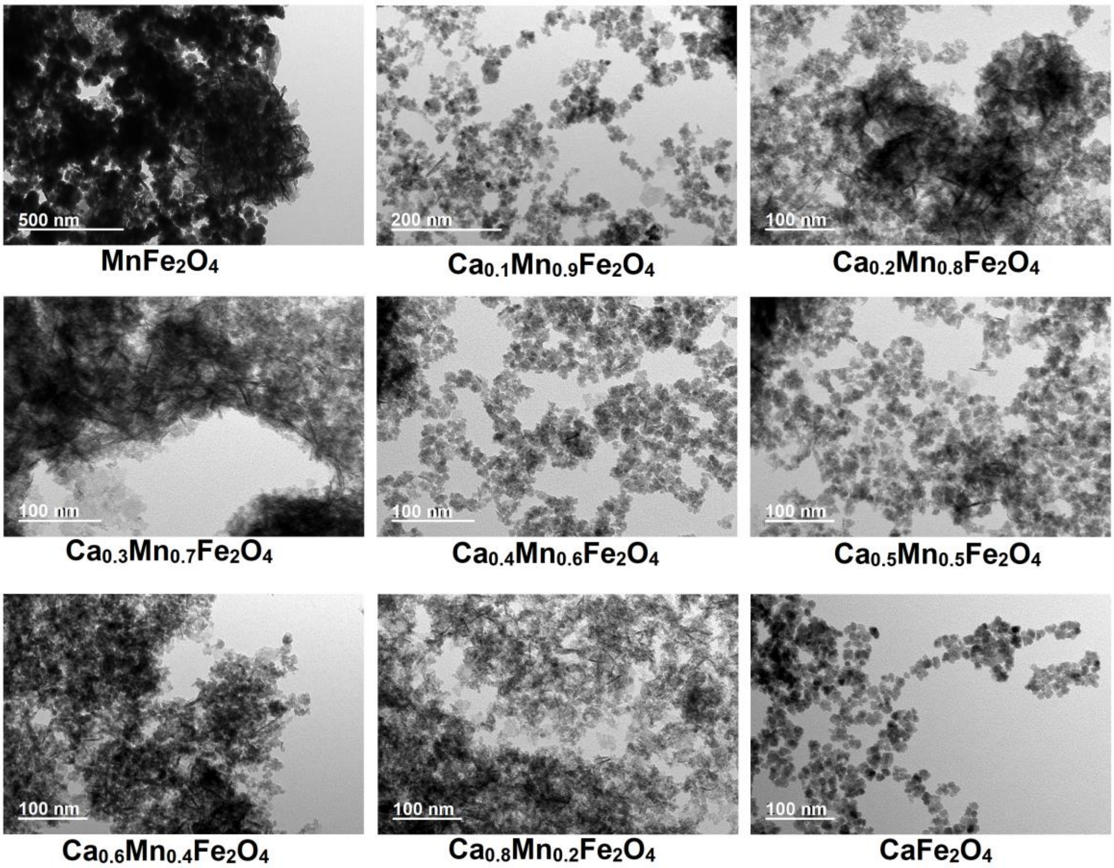

Figure S1. TEM images with more structures obtained in the synthesized calcium and manganese-doped ferrites at 90 °C.

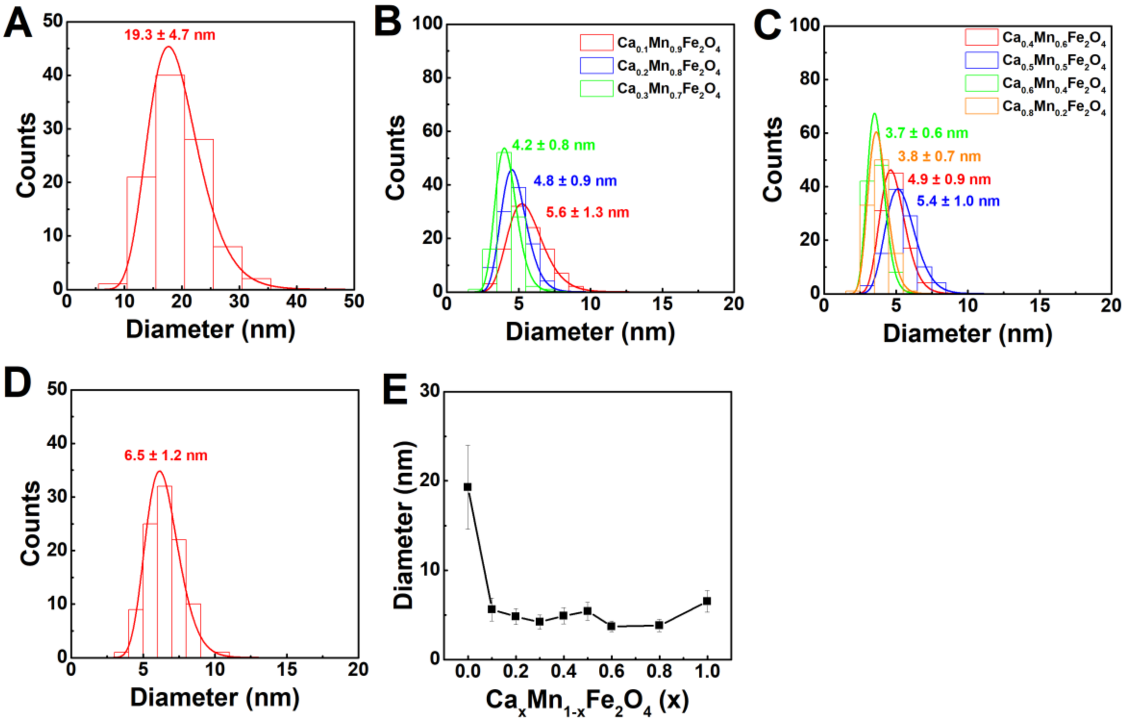

Figure S2. Histograms of the particle sizes measured in the samples of (A) manganese ferrites, (B,C) calcium and manganese-doped ferrites, and (D) calcium ferrites. (E) Dependence of the particle diameter on the nominal composition obtained from the synthesis at 90 °C.

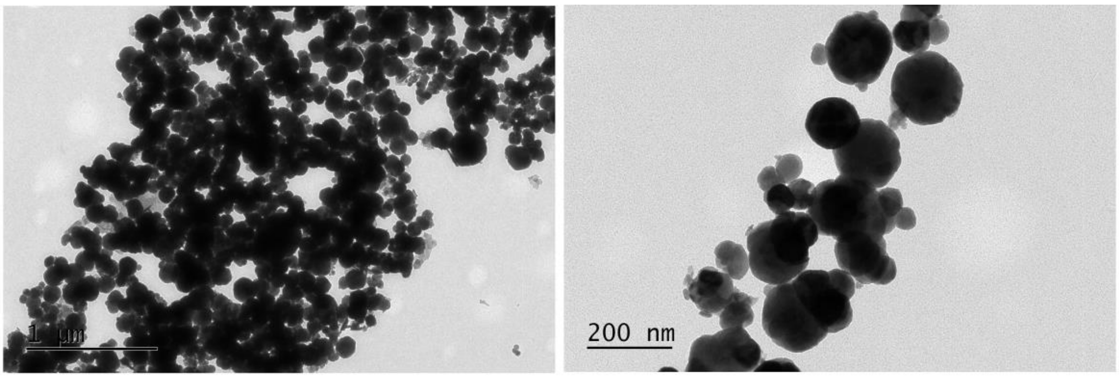

Figure S3. TEM images of manganese ferrites synthesised without citrate at 90 °C.

X-ray diffraction analysis

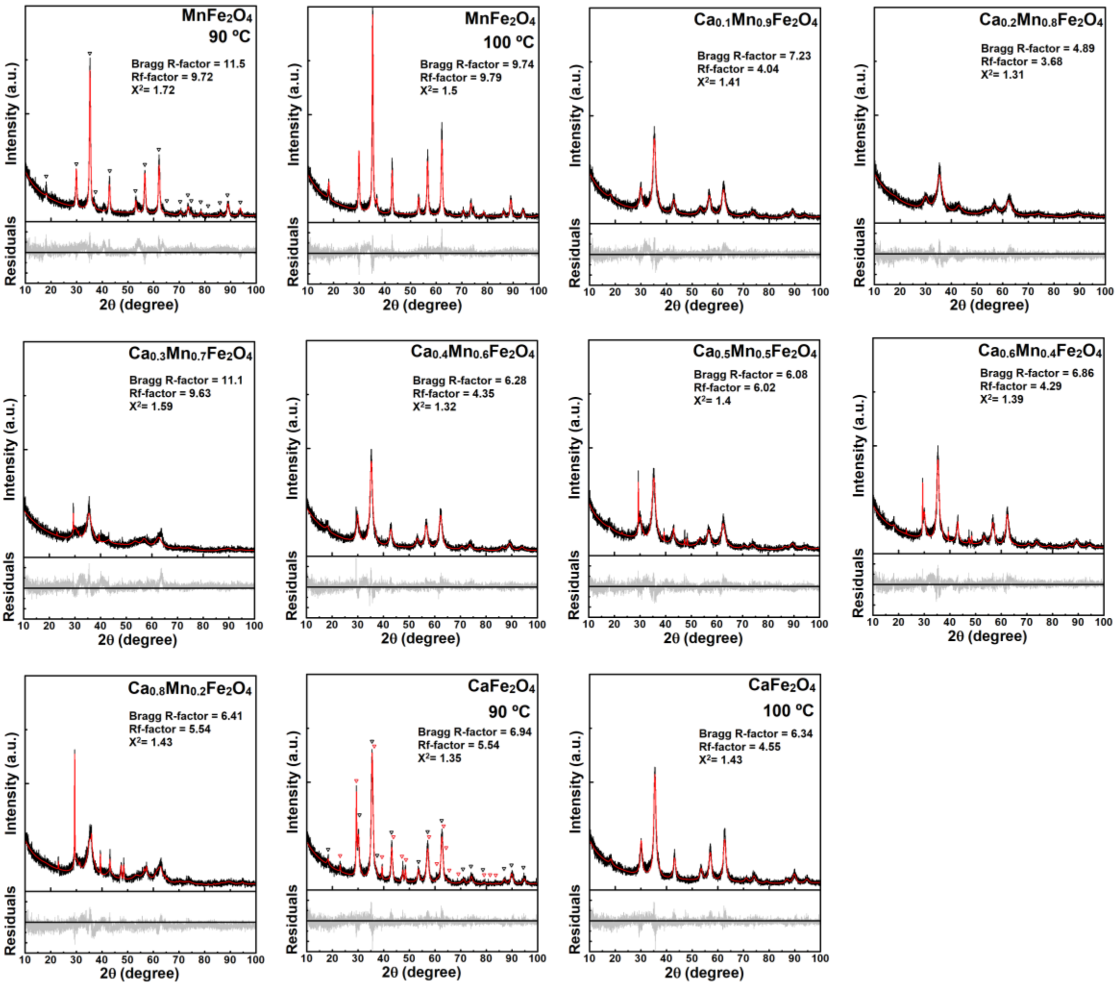

Figure S4. X-ray diffraction patterns of the synthesised nanoparticles (black line) and fitted patterns obtained through Rietveld refinement (red line). The black and red triangles highlight the diffraction pattern contributions from Fd3m (ferrite cubic phase) and R3c (calcite phase) structures.

Table S1. Crystallite size, lattice parameter, X-ray density (d_x), specific surface area in the tetrahedral (L_A) and octahedral (L_B) sites calculated from X-ray profiles for the manganese and calcium-doped ferrites synthesized at 100 °C.

Composition	Crystallite size (nm)	Lattice parameter (*a*) (Å)	d_x (g/cm³)	S×10⁶ (cm²/g)	L_A (Å)	L_B (Å)
$MnFe_2O_4$	14.84	8.44	5.10	0.79	3.65	2.98
$CaFe_2O_4$	5.86	8.38	4.87	2.10	3.63	2.96

Table S2. Tetrahedral (r_A) and octahedral (r_B) radii, tetrahedral bond length (d_{AL}), octahedral bond length (d_{BL}), shared tetrahedral edge (d_{AE}), shared (d_{BE}) and unshared (d_{BEU}) octahedral edges obtained from Rietveld refinement. The oxygen parameters u^{43m} (origin of unit cell considered on an A-site cation) and u^{3m} (origin of unit cell considered at an octahedral vacancy 3m) are also included.

Temperature	Composition	r_A	r_B	d_{AL}	d_{BL}	u^{43m}	u^{3m}	d_{AE}	d_{BE}	d_{BEU}
100 °C	$MnFe_2O_4$	1.76	1.93	1.90	2.07	0.380	0.255	3.10	2.86	2.98
	$CaFe_2O_4$	1.73	1.93	1.86	2.07	0.378	0.253	3.04	2.89	2.96
90 °C	$MnFe_2O_4$	1.78	1.92	1.92	2.06	0.381	0.256	3.14	2.83	2.99
	$Ca_{0.1}Mn_{0.9}Fe_2O_4$	1.72	1.95	1.86	2.09	0.377	0.252	3.04	2.92	2.98
	$Ca_{0.2}Mn_{0.8}Fe_2O_4$	1.71	1.95	1.85	2.08	0.377	0.252	3.03	2.92	2.98
	$Ca_{0.3}Mn_{0.7}Fe_2O_4$	1.66	1.96	1.80	2.10	0.374	0.249	2.94	2.98	2.96
	$Ca_{0.4}Mn_{0.6}Fe_2O_4$	1.74	1.94	1.88	2.08	0.378	0.254	3.07	2.88	2.98
	$Ca_{0.5}Mn_{0.5}Fe_2O_4$	1.68	1.97	1.82	2.11	0.374	0.249	2.96	2.98	2.97
	$Ca_{0.6}Mn_{0.4}Fe_2O_4$	1.74	1.94	1.87	2.08	0.378	0.254	3.06	2.89	2.98
	$Ca_{0.8}Mn_{0.2}Fe_2O_4$	1.66	1.97	1.80	2.11	0.373	0.249	2.94	3.01	2.97
	$CaFe_2O_4$	1.70	1.95	1.83	2.08	0.376	0.251	2.99	2.93	2.96

The increasing content of Ca^{2+} led to a slight increase of octahedral radius and decrease of the tetrahedral site, as well as on the respective bond lengths. In addition, both the shared tetrahedral edge and unshared octahedral edge decreased, while the shared octahedral edge increased. These results can be associated with the migration of the large Ca^{2+} ions to the B-sites.

Regarding the cation distribution, the parameter u^{3m} has a correlation with the degree of inversion [1], in which a value larger than 0.2555 is associated with a normal spinel, while a smaller value is indicative of an inverse spinel. Thus, the particle with a higher amount of Ca^{2+} might display a larger degree of inversion, while the ones with lower content ($x < 0.5$) might have a more disordered or a closer to normal spinel structure. Nonetheless, the close values of u^{3m} to 0.250 is indicative of close to perfect fcc structures [2].

Estimation of cation distribution

The cation distribution was initially estimated through the Bertaut method [3–5]. The method consists on the comparison between diffraction intensities observed in the experimentally obtained diffraction patterns and those calculated for the hypothetical crystal structure. The reflection from the planes (2 2 0), (4 0 0) and (4 4 0) are assumed to be sensitive to the cation distribution. The observed intensity was extracted from the diffraction patterns and the theoretical intensities were calculated by the equation:

$$I_{hkl} = |F|^2_{hkl} p L_p \tag{S1}$$

where F is the structure factor, p is the multiplicity factor, and L_p is the Lorentz polarization factor calculated as:

$$L_p = \frac{1 + \cos^2 2\theta}{\sin^2 \theta \cos \theta} \tag{S2}$$

In general, the observed diffraction intensity ratios were well described by the calculated intensities. Yet, deviations were obtained that can be a result of strain associated with the small size of the nanoparticles affecting the obtained intensities, which might affect the estimated cation distribution. Nevertheless, the results suggest that the Ca^{2+} ions replacing the Mn^{2+} preferentially occupied the octahedral size. In line with the higher lattice parameter at x=0.4, a slight amount of Ca^{2+} migrated to the A-sites at the expense of replacing the Fe^{3+} ions. Regarding the reaction temperature, the synthesis at 100 °C did not produce major changes in the $CaFe_2O_4$ cation distribution, while it strongly affected the degree of inversion in the $MnFe_2O_4$ nanoparticles.

Table S3. Estimated cation distribution from X-ray intensity ratio.

Temperature	x value	Cation distribution		$I_{400/422}$	$I_{220/400}$	$I_{400/422}$	$I_{220/400}$
		**A site**	**B site**	**Obs.**	**Obs.**	**Cal.**	**Cal.**
90 °C	0	$(Mn_{0.1}^{2+}Fe_{0.9}^{3+})$	$[Mn_{0.9}^{2+}Fe_{1.1}^{3+}]$	2.068	1.299	2.057	1.275
	0.1	$(Mn_{0.1}^{2+}Fe_{0.9}^{3+})$	$[Ca_{0.1}^{2+}Mn_{0.8}^{2+}Fe_{1.1}^{3+}]$	2.089	1.329	2.099	1.347
	0.2	$(Ca_{0.03}^{2+}Fe_{0.97}^{3+})$	$[Ca_{0.17}^{2+}Mn_{0.8}^{2+}Fe_{1.03}^{3+}]$	1.966	1.271	1.994	1.316
	0.3	$(Ca_{0.01}^{2+}Fe_{0.99}^{3+})$	$[Ca_{0.29}^{2+}Mn_{0.7}^{2+}Fe_{1.01}^{3+}]$	1.900	1.368	1.907	1.380
	0.4	$(Ca_{0.25}^{2+}Fe_{0.75}^{3+})$	$[Ca_{0.15}^{2+}Mn_{0.6}^{2+}Fe_{1.25}^{3+}]$	2.636	1.172	2.591	1.142
	0.5	$(Ca_{0.14}^{2+}Mn_{0.02}^{2+}Fe_{0.84}^{3+})$	$[Ca_{0.36}^{2+}Mn_{0.48}^{2+}Fe_{1.16}^{3+}]$	2.313	1.365	2.268	1.279
	0.6	$(Ca_{0.14}^{2+}Fe_{0.86}^{3+})$	$[Ca_{0.46}^{2+}Mn_{0.4}^{2+}Fe_{1.14}^{3+}]$	2.125	1.364	2.125	1.365
	0.8	$(Ca_{0.13}^{2+}Mn_{0.09}^{2+}Fe_{0.78}^{3+})$	$[Ca_{0.67}^{2+}Mn_{0.11}^{2+}Fe_{1.22}^{3+}]$	2.047	1.488	2.006	1.424
	1	$(Ca_{0.39}^{2+}Fe_{0.61}^{3+})$	$[Ca_{0.61}^{2+}Fe_{1.39}^{3+}]$	2.667	1.125	2.673	1.142
100 °C	0	$(Mn_{0.7}^{2+}Fe_{0.3}^{3+})$	$[Mn_{0.3}^{2+}Fe_{1.7}^{3+}]$	2.304	1.242	2.283	1.187
	1	$(Ca_{0.23}^{2+}Fe_{0.77}^{3+})$	$[Ca_{0.77}^{2+}Fe_{1.23}^{3+}]$	2.180	1.257	2.214	1.319

Raman spectroscopy of calcium and manganese ferrites

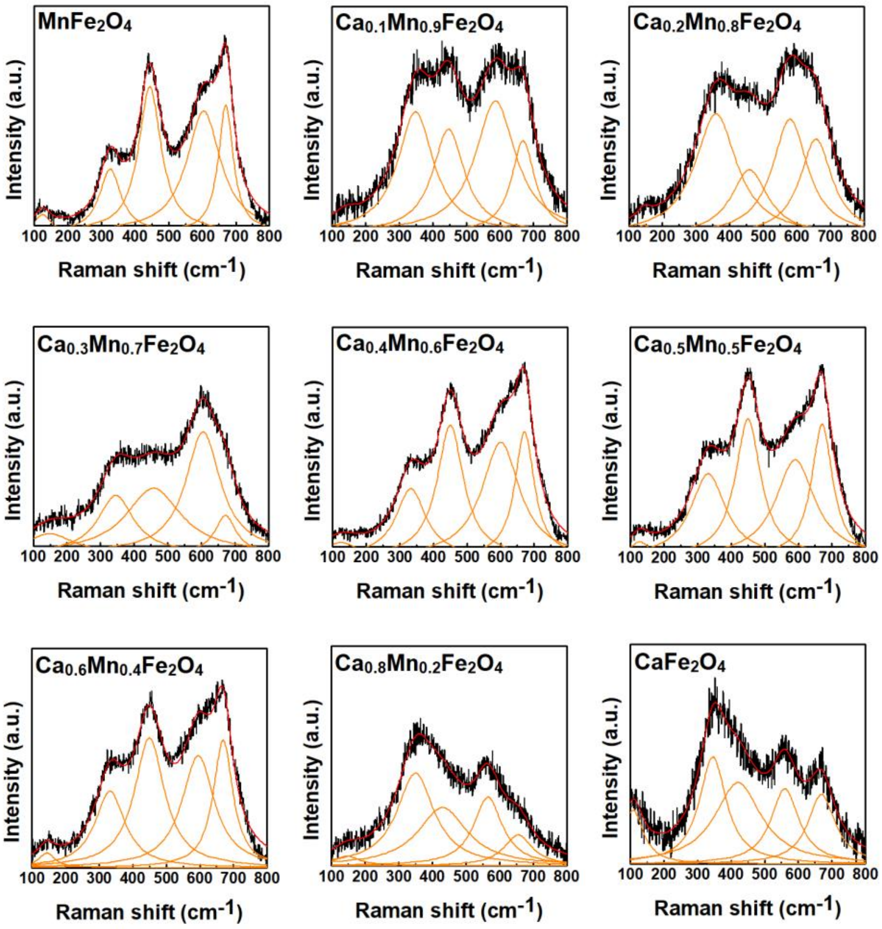

Figure S5. Comparison of the Raman scattering spectra of the manganese- and/or calcium-doped ferrites obtained at 90 °C.

Table S4. Raman shifts (cm^{-1}) obtained from the fitting of Lorentzian bands to the Raman spectra.

Temperature	*x* value	A_{1g}(1)	A_{1g}(2)	T_{2g}(2)	E_g	T_{2g}(1)
	0	670	604	444	326	124
	0.1	669	586	446	348	139
	0.2	657	579	459	359	145
	0.3	671	605	457	345	148
90 °C	0.4	671	600	450	332	124
	0.5	670	590	450	331	127
	0.6	669	594	449	332	144
	0.8	655	565	430	349	145
	1	669	560	420	345	108
100 °C	0	670	605	445	332	121
	1	674	539	447	343	107

The increasing content of Ca^{2+} ions was accompanied with changes in the Raman spectra that closely relate with the dependence of the lattice parameter on its content. For instance, the spectra bands progressively broaden from x = 0 to x = 0.3, which is associated with a decrease of the crystallite size as reported in other calcium-doped ferrites [6]. Concomitantly, there is a steep decrease of the lattice parameter that has been reported to occur for larger Ca^{2+} content [7], and consequently a slight increase of the crystal density. The increase of Ca^{2+} content to x = 0.4 led to an increased particle size and relaxation of the lattice parameter, producing intense and sharper signals like the sample with x = 0. In line with other reports [7], an increase of the lattice parameter occurred for an increased calcium content in the tetrahedral sites. Further increase of the Ca^{2+} ions content is again accompanied with a decrease of the lattice parameter, particle size and crystallite density (reaching the lowest at x = 1), which consequently led to an incremental decrease, red-shift and broadening of the Raman bands. Nonetheless, the general red-shift with the incremental Ca^{2+} content can be associated with its incorporation leading to a smaller effective force constant/effect mass at A and B sites. At the larger calcium concentration, the increase of particle size resulted in sharper Raman band, and an E_g band more intense than the remaining contributions, which has also been reported for other partially inverted metal-doped ferrites [8,9]. Besides, considering that the A_{1g} and T_{2g} bands are mainly associated with the oxygen motion in the A and B sites, respectively, the results further suggest that the increasing Ca^{2+} content from x = 0 to x = 0.3 forces the migration of Fe^{3+} from A to B sites, which is also observed at a Ca^{2+} content of $x = 0.8$ and $x = 1$ and in close qualitative agreement with the estimated cation distribution from XRD.

Optical properties

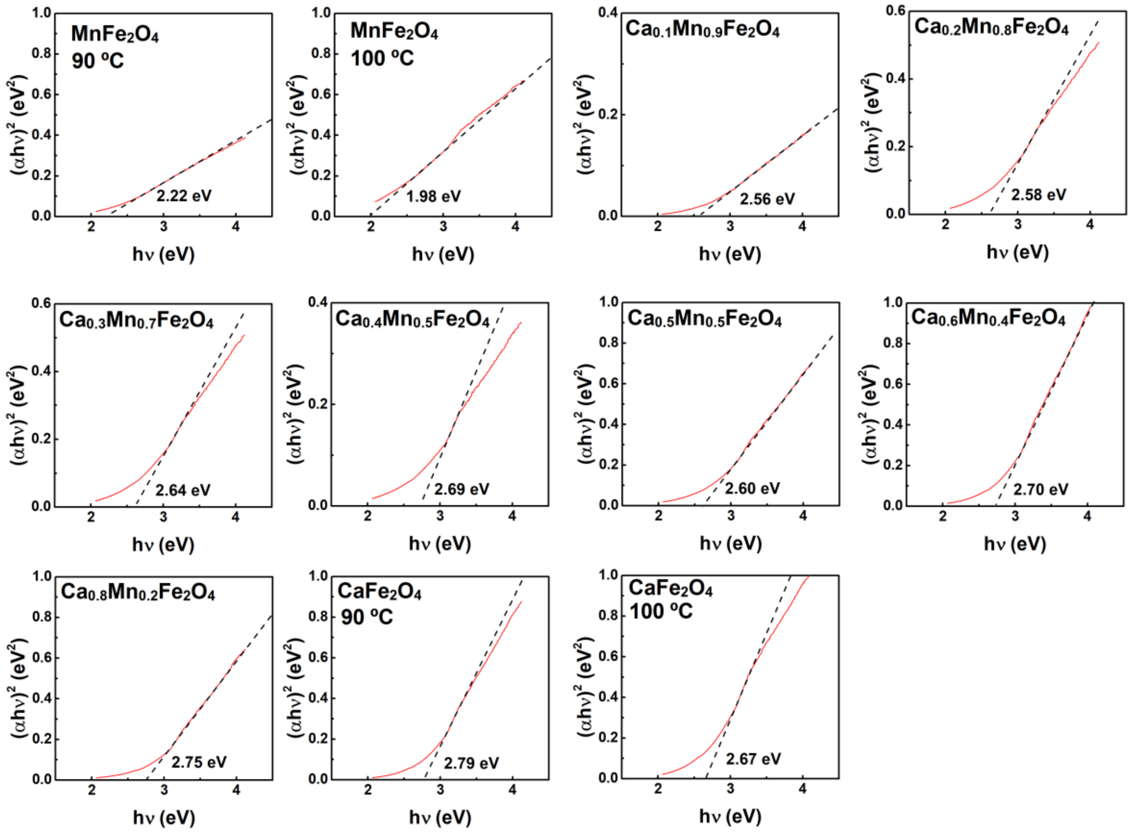

Figure S6. Tauc plots obtained for the manganese- and/or calcium-doped ferrites obtained at 90 °C and 100 °C.

Dynamic light scattering correlograms

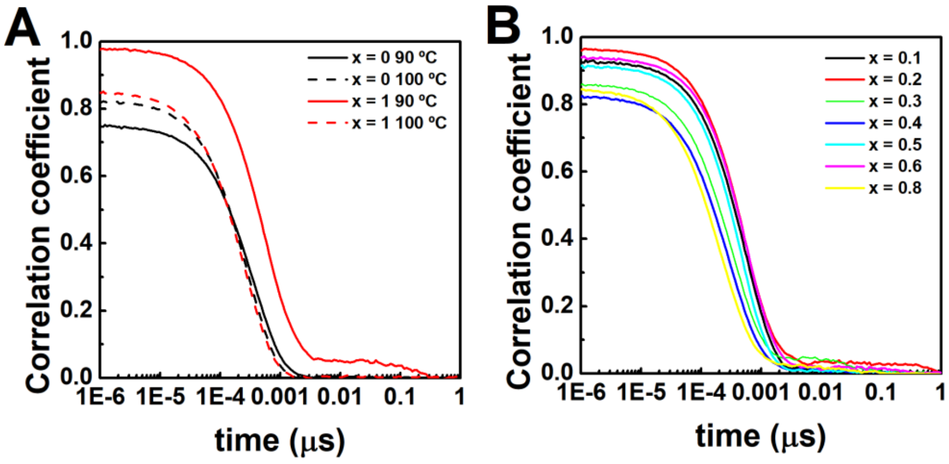

Figure S7. DLS correlograms obtained for the manganese- and/or calcium-doped ferrites obtained at 90 °C (from $x = 0$ to $x = 1$) and 100 °C ($x = 0$ and $x = 1$).

Magnetic properties

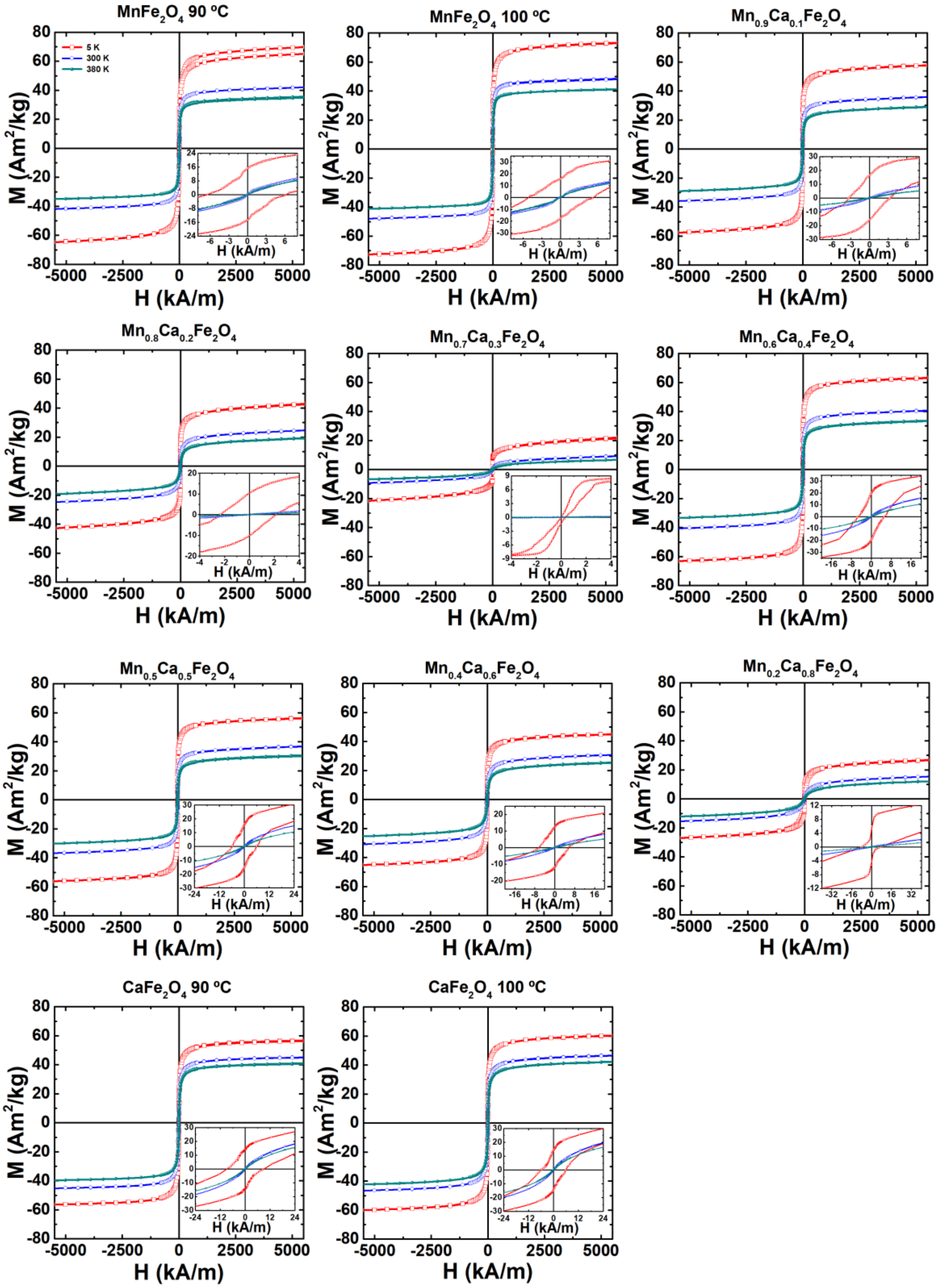

Figure S8. Magnetization dependence on the applied magnetic field obtained for the manganese- and/or calcium-doped ferrites obtained at 90 °C (from x = 0 to x = 1) and 100 °C (x = 0 and x = 1) measured at 5 K, 300 K and 380 K. Inset: Magnetization dependence at lower applied magnetic fields.

Table S5. Experimentally determined magnetic properties measured at 5 K, 300 K and 380 K: saturation magnetization (M_S); remnant magnetization (M_R); squareness (M_R/M_S); coercivity (H_C). In general, the Hc values at 300 K and 380 K were obtained near or below the experimental error, thus being considered meaningless.

Synthesis temperature	*x* value	T (K)	M_S (Am^2/kg)	H_C (kA/m)	M_R (Am^2/kg)	M_R/M_S
90 °C	0	5	70	6.53	15.1	0.2157
		300	42.1	0.32	0.6	0.0143
		380	35.7	0.08	0.1	0.0028
	0.1	5	58	3.26	16.0	0.2759
		300	36.0	0.08	0.2	0.0056
		380	29.3	0.08	0.1	0.0034
	0.2	5	43.0	2.39	9.7	0.2256
		300	24.8	0.16	0.04	0.0016
		380	19.5	0.16	0.02	0.0010
	0.3	5	22.0	0.56	0.8	0.0364
		300	9.4	0.08	0.004	0.0004
		380	6.8	0.08	0.002	0.0003
	0.4	5	63	4.93	19	0.3016
		300	40.8	0.16	0.2	0.0049
		380	33.8	0.16	0.1	0.0030
	0.5	5	45	5.81	12.1	0.2689
		300	30.9	0.16	0.07	0.0023
		380	25.6	0.16	0.04	0.0016
	0.6	5	56	5.89	15.4	0.2750
		300	36.9	0.08	0.2	0.0054
		380	30.8	0.08	0.1	0.0032
	0.8	5	26.9	7.32	5	0.1859
		300	15.5	0.08	0.008	0.0005
		380	12.2	0.08	0.004	0.0003
	1	5	57	8.99	14.1	0.2474
		300	45.2	0.16	0.2	0.0044
		380	41.0	0.08	0.2	0.0049
100 °C	0	5	73	4.85	16.4	0.2247
		300	48.6	0.08	0.2	0.0041
		380	41.4	0.08	0.1	0.0024
	1	5	60.4	5.73	14	0.2318
		300	46.7	0.16	0.2	0.0043
		380	42.4	0.16	0.2	0.0047

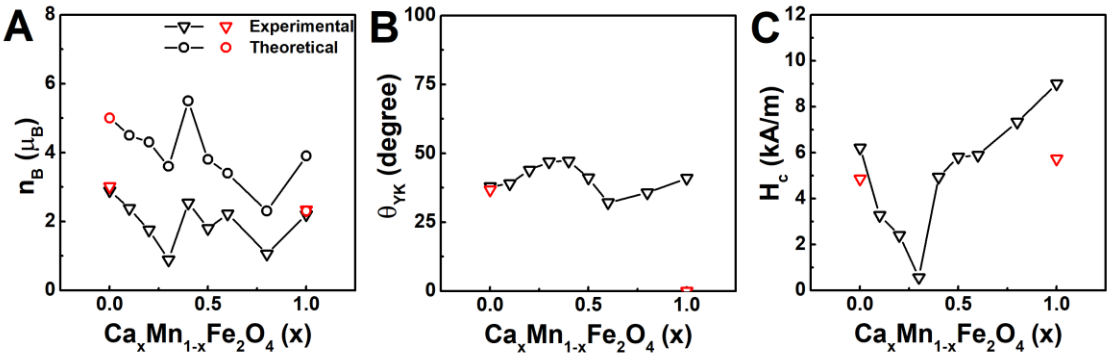

Figure S9. Dependence of (**A**) the experimental (inverted triangle) at 5 K and theoretical (open circles) magnetic moment, (**B**) Yafet-Kittel angle, and (**C**) coercivity measured at 5 K on the Ca/Mn content for the particles synthesised at 90 °C (black tringles) and 100 °C (red triangles).

To assess the impact of the composition and cation distribution on the magnetic properties, the magnetic moment of the particles was initially estimated according to the Néel's two-sublattice model of ferrimagnetic particles. In this model, the magnetic moments of ions on the tetrahedral and octahedral sites are collinear and antiparallel. The theoretical magnetic moment per formula unit μ_B (n_B) was calculated from the XRD estimated cation distribution, and is described as [5,10]:

$$n_B = M_B(x) - M_A(x) \tag{S3}$$

in which $M_A(x)$ and $M_B(x)$ are the A and B sublattice magnetic moments, respectively. The magnetic moment of Ca^{2+} was considered to be 0 μ_B, while both Fe^{3+} and Mn^{2+} were 5 μ_B. The experimental net magnetic moment per formula unit were determined by the relation [11]:

$$n_B = \frac{M_W \times M_S}{5585} \tag{S4}$$

where M_W is the molecular weight, M_S is saturation magnetization, and 5585 is the magnetic factor. The profile of the theoretical particle moments calculated from the estimated XRD cation distribution are in close agreement with the experimental values calculated from the saturation magnetization. Hence, the decrease of the saturation magnetization for a Ca^{2+} content x up to 0.3 can be ascribed to its preferential localization to the B sites, thus reducing the resulting magnetic moment. Further Ca^{2+} increase promoted its distribution to the A sites and forced the migration of Fe^{3+} to the B sites, leading to a strong increase of the magnetic moment, which progressively decreases afterwards owing to the increasing accumulation of Ca^{2+} in the B-sites. When the Ca^{2+} content reaches $x = 1$, its redistribution between to the A sites led to a re-increase of the magnetic moment. Noticeably, the theoretical estimate matches the experimentally obtained magnetic moment of the $CaFe_2O_4$ particles synthesised at 100 °C, which suggest that these particles can be well described by the Néel model. On the other hand, the remaining cation distributions indicated that magnetic ordering might deviate from the Néel-type ordering, in which the B sites magnetic moment was significantly larger than in the A sites. The obtained difference could be related to the small particle size leading to surface and local spin canting effects. Recently, Yafet-Kittel sublattice model has been employed in several works to explain the deviations [5,7,10–12]. In this mode, a weakening of the A-B super-exchange interaction and strengthening of the B-B

interaction leads to the existence of non-collinear B sub-lattices anti-parallel to the A sites magnetic moments, in which the magnetic moment per formula unit μ_B is described as:

$$n_B = M_B(x)\cos\theta_{YK} - M_A(x) \quad \text{(S5)}$$

where θ_{YK} is the Yafet-Kittel angle. The increase of the B-B interaction and consequently, the canting angle, as a result of Fe^{3+} migration to B sites has been suggested for other calcium-doped ferrites [7,13]. Oppositely, an increasing content of Mn^{2+} in substitution of diamagnetic ions (such as Mg^{2+} and Zn^{2+}) has been reported to increase the A-B super-exchange interaction, thus promoting the Néel's type magnetic ordering [14], while an increase has been reported in other ferrites as a result of localization of diamagnetic ions in the A-sites and/or occupation of B-sites by Mn^{2+} [15,16]. In the present work, the experimental $MnFe_2O_4$ magnetic moment did not agree with the Néel model, which suggest that the particles might indeed display canted magnetic moments in the B sites and be better described by the Yafet-Kittel sublattice model. From the cation distribution, the progressive accumulation of Fe^{3+} in the B sites with the Ca^{2+} content x from 0 until 0.4 would be expected to induce a larger canted angle, while the progressive accumulation of Ca^{2+} in the B sites until 0.6 would reduce the B-B interaction and consequently diminish the canted angle. The Ca^{2+} content increase from 0.6 to 1 is again accompanied by migration of Fe^{3+} to the B-sites, thus leading to a re-increase of the canted angle as displayed in figure S9B. Despite the non-linear dependence of the magnetic properties on the Ca^{2+}/Mn^{2+} ratio, the results clearly suggest that the ratio and the synthesis conditions not only influence the size, morphology, and cation distribution, but also the magnetic ordering.

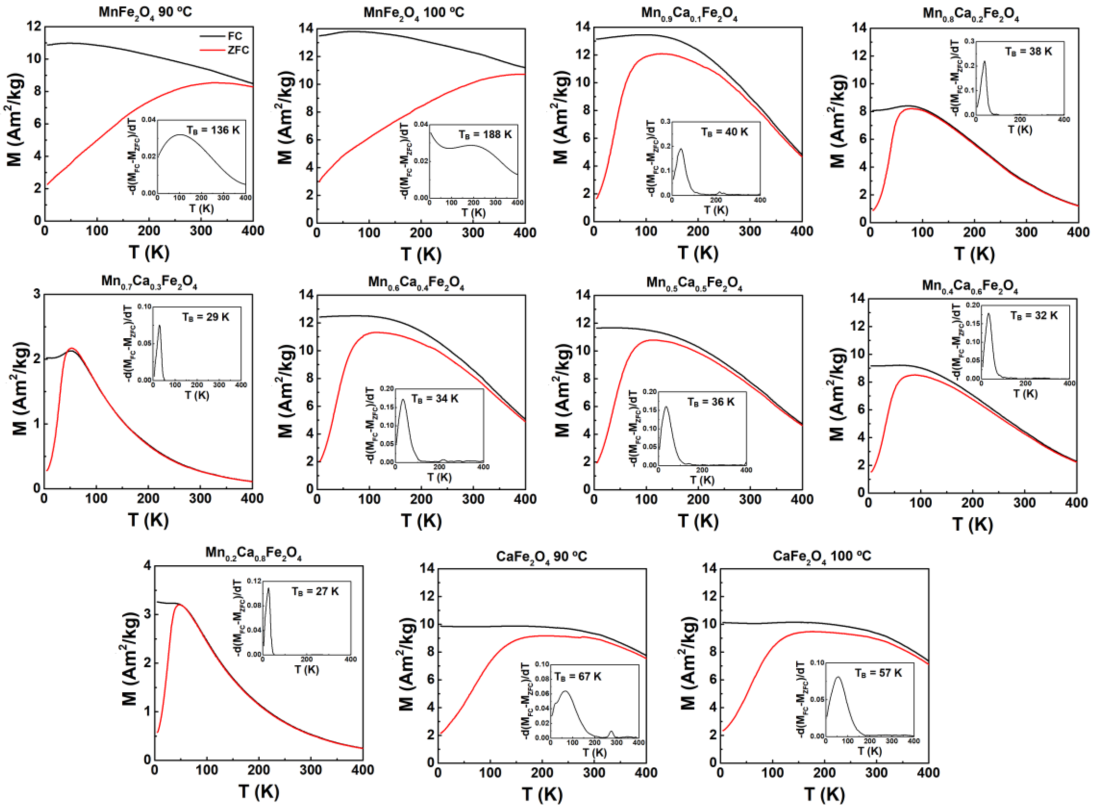

Figure S10. FC and ZFC curves under a field of 7.96 kA/m and calculated blocking temperature obtained for the manganese- and/or calcium-doped ferrites obtained at 90 °C (from $x = 0$ to $x = 1$) and 100 °C ($x = 0$ and $x = 1$).

References

1. Islam, K.; Haque, M.; Kumar, A.; Hoq, A.; Hyder, F.; Hoque, S.M. Manganese Ferrite Nanoparticles ($MnFe_2O_4$): Size Dependence for Hyperthermia and Negative/Positive Contrast Enhancement in MRI. *Nanomaterials* **2020**, *10*, 1–23, doi:10.3390/nano10112297.

2. Satalkar, M.; Kane, S.N. On the Study of Structural Properties and Cation Distribution of $Zn_{0.75-x}Ni_xMg_{0.15}Cu_{0.1}Fe_2O_4$ Nano Ferrite: Effect of Ni Addition. *J. Phys. Conf. Ser.* **2016**, *755*, 012050, doi:10.1088/1742-6596/755/1/012050.

3. Babu, K. V.; Kumar, G.V.S.; Satyanarayana, G.; Sailaja, B.; Lakshmi, C.C.S. Microstructural and Magnetic Properties of $Ni_{1-x}Cu_xFe_2O_4$ (x = 0.05, 0.1 and 0.15) Nano-Crystalline Ferrites. *J. Sci. Adv. Mater. Devices* **2018**, *3*, 236–242, doi:10.1016/j.jsamd.2018.04.003.

4. Weil, L.; Bertaut, F.; Bochirol, L. Propriétés Magnétiques et Structure de La Phase Quadratique Du Ferrite de Cuivre. *J. Phys. le Radium* **1950**, *11*, 208–212, doi:10.1051/jphysrad:01950001105020800.

5. Nikam, D.S.; Jadhav, S. V.; Khot, V.M.; Bohara, R.A.; Hong, C.K.; Mali, S.S.; Pawar, S.H. Cation Distribution, Structural, Morphological and Magnetic Properties of $Co_{1-x}Zn_xFe_2O_4$ (x = 0-1) Nanoparticles. *RSC Adv.* **2015**, *5*, 2338–2345, doi:10.1039/c4ra08342c.

6. Galinetto, P.; Albini, B.; Bini, M.; Mozzati, M.C. Raman Spectroscopy in Zinc Ferrites Nanoparticles. In *Raman Spectroscopy*; Nascimento, G., Ed.; IntechOpen: London, 2018.

7. Bamzai, K.K.; Kour, G.; Kaur, B.; Kulkarni, S.D. Preparation, and Structural and Magnetic Properties of Ca Substituted Magnesium Ferrite with Composition $MgCa_xFe_{2-x}O_4$ (x = 0.00, 0.01, 0.03, 0.05, 0.07). *J. Mater.* **2014**, *2014*, 184340, doi:10.1155/2014/184340.

8. Wang, Z.; Schiferl, D.; Zhao, Y.; O'Neill, H.S.C. High Pressure Raman Spectroscopy of Spinel-Type Ferrite $ZnFe_2O_4$. *J. Phys. Chem. Solids* **2003**, *64*, 2517–2523, doi:10.1016/j.jpcs.2003.08.005.

9. Freire, R.M.; Ribeiro, T.S.; Vasconcelos, I.F.; Denardin, J.C.; Barros, E.B.; Mele, G.; Carbone, L.; Mazzetto, S.E.; Fechine, P.B.A. $MZnFe_2O_4$ (M = Ni, Mn) Cubic Superparamagnetic Nanoparticles Obtained by Hydrothermal Synthesis. *J. Nanoparticle Res.* **2013**, *15*, 1616, doi:10.1007/s11051-013-1616-3.

10. Kurian, J.; Lahiri, B.B.; Mathew, M.J.; Philip, J. High Magnetic Fluid Hyperthermia Efficiency in Copper Ferrite Nanoparticles Prepared by Solvothermal and Hydrothermal Methods. *J. Magn. Magn. Mater.* **2021**, *538*, 168233, doi:10.1016/j.jmmm.2021.168233.

11. Topkaya, R.; Baykal, A.; Demir, A. Yafet–Kittel-Type Magnetic Order in Zn-Substituted Cobalt Ferrite Nanoparticles with Uniaxial Anisotropy. *J. Nanoparticle Res.* **2013**, *15*, 1359, doi:10.1007/s11051-012-1359-6.

12. Ouyahia, S.; Rais, A.; Bozzo, B.; Taibi, K.; Addou, A. Cations Distribution by Rietveld Refinement and Magnetic Properties of $MgCr_xFe_{2-x}O_4$ Spinel Ferrites. *Appl. Phys. A Mater. Sci. Process.* **2020**, *126*, 1–9, doi:10.1007/s00339-020-03865-z.

13. Assar, S.T.; Abosheiasha, H.F. Effect of Ca Substitution on Some Physical Properties of Nano-Structured and Bulk Ni-Ferrite Samples. *J. Magn. Magn. Mater.* **2015**, *374*, 264–272, doi:10.1016/j.jmmm.2014.08.011.

14. Sharma, R.; Thakur, P.; Kumar, M.; Barman, P.B.; Sharma, P.; Sharma, V. Enhancement in A-B Super-Exchange Interaction with Mn Substitution in Mg-Zn Ferrites as a Heating Source in Hyperthermia Applications. *Ceram. Int.* **2017**, *43*, 13661–13669, doi:10.1016/j.ceramint.2017.07.076.

15. Nitika; Rana, A.; Kumar, V. Evaluation of Structural, Magnetic, Optical, Electrical, and Humidity Sensing Properties of Manganese-Substituted Zinc Ferrite Nanoparticles. *Appl. Phys. A* **2021**, *127*, 860, doi:10.1007/s00339-021-05016-4.

16. Kumar, P.; Pathak, S.; Singh, A.; Jain, K.; Khanduri, H.; Wang, L.; Kim, S.-K.; Pant, R.P. Observation of Intrinsic Fluorescence in Cobalt Ferrite Magnetic Nanoparticles by Mn^{2+} Substitution and Tuning the Spin Dynamics by Cation Distribution. *J. Mater. Chem. C* **2022**, *10*, 12652–12679, doi:10.1039/D2TC02605H.
